# Exon-Level Transcriptome Profiling in Murine Breast Cancer Reveals Splicing Changes Specific to Tumors with Different Metastatic Abilities

**DOI:** 10.1371/journal.pone.0011981

**Published:** 2010-08-06

**Authors:** Amandine Bemmo, Christel Dias, April A. N. Rose, Caterina Russo, Peter Siegel, Jacek Majewski

**Affiliations:** 1 Department of Human Genetics, McGill University, Montreal, Quebec, Canada; 2 Department of Medicine, McGill University, Montreal, Quebec, Canada; 3 Department of Biochemistry, McGill University, Montreal, Quebec, Canada; 4 Department of Anatomy and Cell Biology, McGill University, Montreal, Quebec, Canada; 5 McGill University and Genome Quebec Innovation Center, Montreal, Quebec, Canada; Baylor College of Medicine, United States of America

## Abstract

**Background:**

Breast cancer is the second most frequent type of cancer affecting women. We are increasingly aware that changes in mRNA splicing are associated with various characteristics of cancer. The most deadly aspect of cancer is metastasis, the process by which cancer spreads from the primary tumor to distant organs. However, little is known specifically about the involvement of alternative splicing in the formation of macroscopic metastases. Our study investigates transcript isoform changes that characterize tumors of different abilities to form growing metastases.

**Methods and Findings:**

To identify alternative splicing events (ASEs) that are associated with the fully metastatic phenotype in breast cancer, we used Affymetrix Exon Microarrays to profile mRNA isoform variations genome-wide in weakly metastatic (168FARN and 4T07) and highly metastatic (4T1) mammary carcinomas. Statistical analysis identified significant expression changes in 7606 out of 155,994 (4%) exons and in 1725 out of 189,460 (1%) intronic regions, which affect 2623 out of 16,654 (16%) genes. These changes correspond to putative alternative isoforms—several of which are novel—that are differentially expressed between tumors of varying metastatic phenotypes. Gene pathway analysis showed that 1224 of genes expressing alternative isoforms were involved in cell growth, cell interactions, cell proliferation, cell migration and cell death and have been previously linked to cancers and genetic disorders. We chose ten predicted splice variants for RT-PCR validation, eight of which were successfully confirmed (MED24, MFI2, SRRT, CD44, CLK1 and HNRNPH1). These include three novel intron retentions in CD44, a gene in which isoform variations have been previously associated with the metastasis of several cancers.

**Conclusion:**

Our findings reveal that various genes are differently spliced and/or expressed in association with the metastatic phenotype of tumor cells. Identification of metastasis-specific isoforms may contribute to the development of improved breast cancer stage identification and targeted therapies.

## Introduction

In breast cancer patients, tumor metastases at distant sites are the main cause of death [1]. However, the molecular mechanisms of metastasis of breast cancer remain unclear. It is thought that changes occurring at the level of RNA processing contribute to cancer. Alternative splicing (AS) of pre-mRNA, a key post-transcriptional mechanism allowing for the production of distinct proteins from a single gene, affects over 90% of human genes [2], [3]. Such splicing events are responsible for generating mRNAs that encode protein isoforms that can have very different biological properties and functions. A well-studied example is the BCL-X gene, whose two major transcript isoforms produce two proteins having antagonistic functions [4]: the short form (BCL-XS) promotes apoptosis while the long form (BCL-XL) is anti-apoptotic. Moreover, overexpression of BCL-XL has been reported to enhance the metastatic potential of breast tumor cells in patients [5]. Another interesting example is CD44, a multi-functional cell adhesion protein for which many splice variants have been associated with the growth and progression of multiple tumor types [6], [7], [8]. CD44 shows an unusual pattern of splice variants in mammary tumorigenesis, which arises from the differential usage of 10 internal exons. In breast cancer metastases, CD44 isoforms with variable inclusion of these 10 exons are expressed; whereas preneoplasias show a more restricted exon inclusion pattern [9]. The CD44v5 isoform has been identified to enhance tumor cell invasiveness [10], and tumor cells expressing the CD44v4-10 variant form larger volume primary tumors and more metastases compared to tumors expressing wild-type CD44 [11]. Moreover, changes in splicing during cancer appear to alter cell morphology, adhesion, migration, apoptosis and proliferation processes which are hallmarks of metastasis [12]. Cancer metastasis is a progressive process and thus transcript isoforms associated with specific metastatic phenotype may serve as potential biomarkers for aggressive disease and may be relevant in the development of targeted treatment strategies of breast cancer patients.

The Affymetrix GeneChip Exon 1.0 ST (Exon Array), a tool for exon-based transcriptome profiling, can be used to detect differences in isoform-level expression and has been applied successfully in several studies [13], [14], [15]. It allows the expression profiling of over a million individual exons, both those that are known and predicted. In this study, we used this technology in a murine breast cancer model to identify changes in splicing that are associated to metastatic phenotypes. We simultaneously analyzed exon and transcript expression in tumor tissues derived from three murine mammary carcinoma cell lines (168FARN, 4T07 and 4T1), each possessing different metastatic phenotypes [16]. The criterion used to assign the metastatic potential of these cell lines is based on their ability to form lung metastases. 168FARN tumor cells can be detected in lymph nodes but fail to extravasate and are rarely detected in the lungs. Cells from 4T07 tumors reach the lung via the blood, but are unable to develop into macroscopic metastatic nodules. Lastly, 4T1 cells possess the ability to spontaneously metastasize to distant sites, including the lung, bone and liver [17]. By performing statistical analysis of the Exon Array data, transcript isoforms associated with metastatic phenotypes of these different tumors were identified. Nearly 16% of genes display at least one differently expressed exon across the panel of murine breast tumors. Additionally, gene pathway profiling was performed with candidate genes using the Ingenuity Pathway Analysis software. Half of these genes are known to be associated with cancer hallmarks and genetic disorders, while the remaining candidates have no established link with cancer progression and metastasis and may constitute novel candidates for metastasis diagnosis and therapy.

## Results

To seek transcriptome changes at the exon scale during metastasis progression, we measured global exon expression in murine breast tumors that have differential metastatic behaviors. The breast carcinoma cell lines were individually injected into the mouse mammary fat pads as previously described [18] and total RNA was purified from the resulting tumors once they reached a volume between 100 and 125 mm^3^. Samples were then labeled and hybridized to GeneChip Mouse 1.0 ST arrays that provide multiple probes (probe sets) per exon and target over a million known and predicted exons. The microarray data has been deposited in the Gene Expression Omnibus Database (accession: GSE21994). We analyzed the full probe set annotation (493,710 probe sets), interrogating 345,454 exons and introns. In addition to the core probe sets, we extended our analysis to the non-core probe sets, which are those supported by EST and predictive evidence not present within RefSeq and full-length mRNA GenBank records. Concurrent with exome profiling, we estimated the gene-level intensity by summarizing, for each gene, the signal values of all the probe sets belonging to the gene (meta-probe set). Hence, the full dataset was summarized into 16,654 unique well-annotated transcripts. We applied several filtering steps to discard genes and exons with expression values close to background in an effort to minimize the false positive rate (see Methods). We obtained 183,610 expressed probe sets, corresponding to 137,950 exons or introns belonging to 11,082 genes. To determine differently expressed exons and genes across tumor samples, we simultaneously performed either a one-way ANOVA test on probe set logarithmic intensities or a one-way ANOVA test on meta-probe set logarithmic intensities. At the probe set level, we performed two concurrent analyses: a probe set expression-intensity analysis and a probe set gene-level normalized intensity analysis. The gene-level normalized intensity is the ratio of the probe set expression intensity to the expression intensity of the meta-probe set that the probe set belongs to. The splicing index (SI) for a probe set is then defined as the ratio of gene-level normalized intensities in one sample relative to another. Subsequent to ANOVA tests, we applied a 0.05-level FDR (False discovery rate) correction to determine the P-value threshold to identify significant exons (P<6.36×10^−4^ for the probe set expression analysis; P<7.10×10^−4^ for the SI analysis) and the significance of whole gene expression (P<8.46×10^−3^: over-expressed in a sample). Differentially expressed exons between paired samples were located by performing pairwise T-test comparisons (168FARN and 4T07 against 4T1). The log_2_ transformed expression fold-changes (4T07/4T1 and 168FARN/4T1) between paired sample comparisons were also computed.

### Gene expression and isoform variations specific to tumor metastatic phenotype

**Figure 1:**
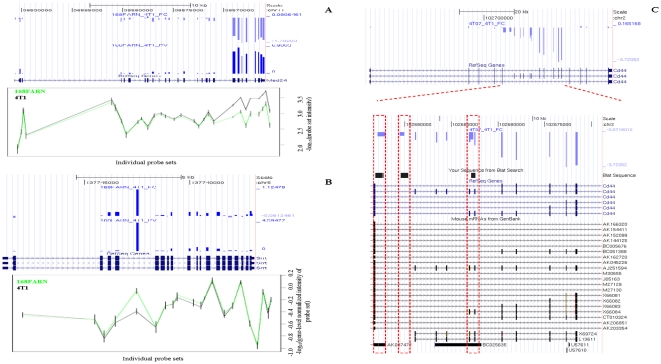
Examples of visualization of gene expression patterns showing isoform variations. A: Visualization of the expression pattern of MED24 gene showing an alternative start in 4T1. In the top panel, the horizontal scale corresponds to each probe set within the gene from the 5′ to 3′ ends. The blue bars indicate the comparison between 168FARN and 4T1 samples. From top to bottom we plotted the log_2_(fold-change) in expression, between the samples compared, and the statistical significance, −log_10_(p-value). The bottom panel shows the log_10_(expression intensity) of individual probe sets (from the top panel) in samples 168FARN and 4T1. Note that the seven last probe sets and the 3′ UTR region that are over-expressed only in 4T1 indicate an additional isoform in 4T1 starting from exon 20 start. B: Visualization of the expression pattern of SRRT gene showing an intron inclusion. In the top panel, the horizontal scale corresponds to each probe set within the gene from the 5′ to 3′ ends. The blue bars indicate the comparison between 168FARN and 4T1 samples. From top to bottom we plotted the the log_2_(fold-change) in gene-level normalized intensity between the samples compared and the statistical significance, −log_10_(p-value). The bottom panel shows the log_10_(gene-level normalized intensity) of individual probe sets (from the top panel) in samples 168FARN and 4T1. We note an intron inclusion between exons 5 and 6 in samples 168FARN. C: Visualization of the expression pattern of CD44 gene showing several internal cassette exons and three novel intron inclusions. In the top panel, the horizontal scale corresponds to each probe set within the gene from the 5′ to 3′ ends. The blue bars indicate the comparison between 4T07 and 4T1. From top to bottom we plotted the log_2_(fold-change) in expression, between the samples compared. The bottom panel shows a close-up of the CD44 region containing the differentially expressed exons and introns. The first custom track displays the fold-changes and the second custom track displays the sequencing alignment of the three retained introns. We note that in this example, exons 8, 11 and 13, two intronic sequences between exons 5 and 6, and one intronic sequence between exons 9 and 10, are over-expressed in the 4T1 sample.

We obtained 10,744 probe sets targeting 9331 (2.7%) introns or exons showing significant expression changes belonging to 2623 (15.7%) genes. Of these, 1772 (10.6%) genes displayed expression changes at the whole transcript level while 851 (5.1%) showed isoform changes without corresponding whole gene expression changes. To visualize ASEs in the context of EST/mRNA or genome annotation, we uploaded our data as a track in the UCSC Genome Browser [19]. For each gene, we plotted the paired T-test P-values and the expression fold-changes of each individual exon (examples shown in Figure 1). Using this visualization, we manually curated the results and selected 203 genes (143 from the expression intensities analysis and 60 from the SI analysis; Details reported in Supplementary File S1 and Supplementary File S2) for which the transcript variation pattern was clearly interpretable and confidently classifiable into the gene expression change category or into the standard ASE categories. These 203 candidates showed evidence for differential promoter usage, polyadenylation site usage, ASE and whole gene expression changes. We calculated the proportion of each isoform variation type among our classified candidate genes, which revealed that 26.1% of these genes showed whole gene expression changes with some of them showing additional splicing changes. A large proportion of genes showed only isoform changes (examples in Table 1), namely intron inclusion or inclusion of cryptic, unannotated exons (46.4%) and cassette exon usage (13.5%). Furthermore, 7.2% of isoform changes occurred at the level of transcript initiation or transcript termination. Thirteen genes showed changes within the UTR regions: three genes had differential 5′ UTR changes and 10 presented 3′ UTR changes. We found only one gene showing an alternative 5′ splice site. However, the remaining significant genes (2420) presented complex variation patterns that were difficult to categorize. Below, we describe some potentially interesting examples detected by the analysis.

**Table 1:** List of some alternatively expressed probe sets.

Gene name^1^	PS^2^	PS location^3^	168FARN vs. 4T1		4T07 vs. 4T1		ASE^6^	Evidence^7^
			P-value^4^	FC^5^	P-value	FC		
CD44	4534496	E13	3.14×10^−04^	2.81	3.38×10^−04^	2.64	CE	Yes
	4740112	E11	6.39×10^−04^	2.77	6.39×10^−04^	2.72	CE	Yes
	4461784	I (E9-E10)	1.94×10^−04^	2.06	1.55×10^−03^	1.46	CE	No
	5425762	E8	1.02×10^−05^	1.50	2.42×10^−04^	0.96	CE	Yes
	4423264	I (E5-E6)	3.61×10^−05^	1.51	2.28×10^−03^	0.82	II	No
	4622064	I (E5-E6)	9.18×10^−05^	1.18	8.16×10^−03^	0.52	II	No
Itgb1	5044002	I (E9-E10)	2.00×10^−05^	1.58	1.12×10^−03^	0.89	II	Yes
Slc25a29	4968317	3′ UTR	9.56×10^−05^	−2.17	9.56×10^−05^	−2.15	3′ UTR	Yes
MAPK14	4487560	I (E2-E3)	1.47×10^−01^	−0.18	2.05×10^−04^	−0.79	II	No
Msx1	4993066	3′ UTR	2.81×10^−03^	0.88	5.22×10^−04^	1.19	3′ UTR	No
Srrt	5382632	I (E5-E6)	2.54×10^−05^	−1.12	7.90×10^−06^	−1.36	II	No
MFi2	5508279	E13	1.25×10^−04^	−1.88	1.54×10^−06^	−3.37	CE	No

I(Ex-Ey): Intron between exon x and exon y.

The gene name^1^, the probe set ID^2^ and the relative probe set location^3^ in the gene are indicated. For each pairwise comparison, the T-test p-value^4^ and the log_2_(fold-change)^5^ are given. The nature of the isoform change^6^ is shown (CE: cassette exon, II: intronic sequence inclusion, 3′ UTR: differential 3′ UTR). An existing RefSeq, mRNA, or EST supporting the event is also mentioned^7^.

The process of pre-mRNA splicing is regulated by a large number of “splicing-factors” and the misregulation of such genes may be particularly detrimental to many downstream splicing events. HNRNPH1 and CLK1, two trans-acting splicing regulator factors, are predicted by our computational analysis to be differentially spliced between tumor cells. HNRNPH1 is a member of the hnRNP family and retains an intronic sequence between exons 9 and 10 in 168FARN and 4T07 mammary tumors compared to 4T1 tumors. This splice-form causes a reading frameshift and a protein truncation. The hnRNP proteins are required for pre-mRNA processing and maturation and bind to newly synthesized RNA in the nucleus until they are exported to the cytoplasm. Interestingly, a frameshift mutation in HNRNPH1 has been previously identified in gastric cancer [20]. The second example, CLK1, shows a retained intronic sequence in 4T1-derived mammary tumors, situated between exons 5 and 6. This intron retention leads to a reading frameshift and the disruption of a kinase domain in the protein product. This gene codes for a member of the CDC2-like family. Expressed in the nucleus, this protein phosphorylates other serine/arginine-rich (SR) proteins, which has been show to be involved in the regulation of splice site selection during pre-mRNA maturation [21].

We also found isoform variants of several genes involved in cell growth, cell movement, cell proliferation and apoptosis, including CD44, PHB and MAPK14. CD44 isoform variations in cancer have been associated with the metastatic ability of tumor cells, being involved in numerous processes, including cell proliferation, adhesion and invasion [22]. This protein increases the adhesion and invasion of breast cancer cells [7], [23] and decreases cell death and apoptosis of colon cancer cells [24]. We identified a novel isoform of CD44 showing retention of intronic sequences with no previous RefSeq or full cDNA evidence in publicly-available sequence databases (Figure 1C). In this variant, two introns – one between between exons 5 and 6 and the other between exons 9 and 10 were retained in 4T1-derived fully metastatic mammary tumors. These result in reading frame-shifts in the transcript. We also noted a high inclusion rate of exons 8, 11 and 13 within CD44 from 4T1-derived tumors compared to 168FARN and 4T07-derived tumors.

Another example includes PHB-exon 4, which was found to be predominantly expressed in 4T1-derived mammary tumors compared to those arising from the injection of 168FARN or 4T07 cells. The lack of PHB-exon 4 caused a reading frameshift and the removal of 26% of SPFH, an integral membrane domain. Prohibitin (PHB) is an evolutionary conserved gene that is highly expressed in different tissues. It is a cell proliferation regulator and also a tumor suppressor gene. It has been shown that the silencing of prohibitin function increases tumor cell cycle progression in prostate cancer [25]. PHB protein decreases colony formation of T47D breast cancer cells by repressing E2F, a complex of growth regulatory proteins [26]. Moreover, mutations in PHB gene have been associated with sporadic breast cancer [27].

Finally, we found that within MAPK14, preferential retention of intron 2 occurred specifically in 4T07-derived mammary tumors. MAPK14-intron 2 retention shifts the reading frame and disrupts a kinase domain in the protein product. MAPK14 is a member of the MAPK complex whose controlled regulation plays a part in cell proliferation and differentiation, whereas uncontrolled activation can lead to oncogenesis [28].

Strikingly, we also observed some novel transcript isoforms that were not previously annotated. Representative examples are MED24 and SRRT which were subsequently confirmed by qRT-PCR (see below). Our exon array data showed an alternative start of MED24 with the last seven exons of the transcript predominantly expressed in 4T1-derived tumors (Figure 1A). The wild-type MED24, expressed in all our samples, encodes a subunit of the mediator complex TRAP, a transcriptional coactivator complex necessary for the expression of almost all genes. The truncated isoform found in 4T1-derived tumors could potentially create a protein with deleterious activities. Additionally, an intronic sequence in SRRT located between exons 5 and 6 was differentially expressed between tumor samples. This intron showed a strong over-expression in 168FARN and 4T07 cells when comparing to 4T1 mammary tumor cells. SRRT, also known as ARS2, is important for miRNA biogenesis during miRNA-mediated gene silencing in proliferating cells [29].

### Non-coding genic regions are widely retained in breast cancer metastasis

**Figure 2:**
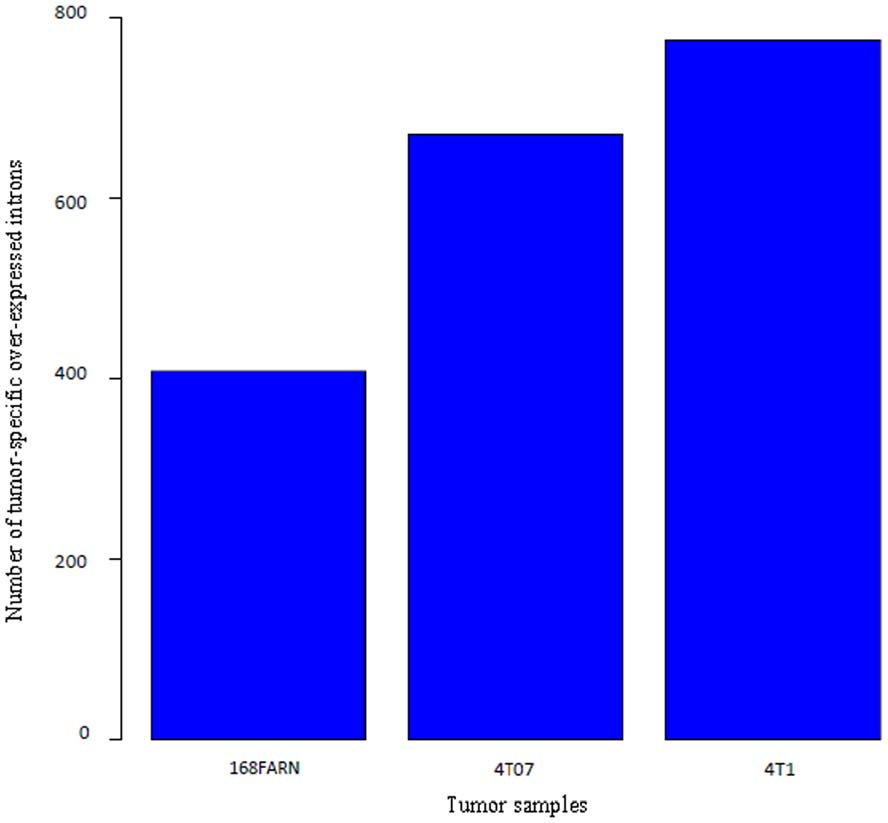
Proportions of tumor-specific over-expressed intronic regions. The tumor samples are plotted on the x-axis, and the number of tumor-specific intron inclusion on the y-axis.

A large number of the differences we detected are represented by non-core probe sets, which targeted intronic regions. We determined that 277,346 of the 493,710 (56.2%) analyzed probe sets were non-core and we decided to interrogate 189,460 intronic regions.We note that 49,588 of these non-core probe sets satisfied the expression filtering criteria, including 2037 which showed expression variations between tumor samples and mapping to 1725 intronic regions. This proportion represents 18% of the total statistically significant introns and exons obtained. Besides the expression variations of known coding regions, cancer cells are susceptible to express such predominantly non-coding regions because of general misregulation of gene expression and splicing. Therefore, the inclusion of non-core probe sets in our analysis was relevant. This enabled us to enrich the novel ASE proportion, mainly the intron retention/cryptic category. Remarkably, the proportion of tumor-specific over-expressed introns (Figure 2) showed that 4T1 mammary tumors, which represent the highest metastatic potential, had the greatest degree of intron inclusion (775 over-expressed intronic regions) followed by 4T07-derived mammary tumors and finally 168FARN samples (671 and 408 over-expressed intronic regions, respectively). However, probe sets outside of annotated transcripts (518,024 probe sets) were excluded from our investigation. We note that some of these excluded probe sets, representing about half of total probe sets on the array, were differentially expressed between tumors. They may form new genes or produce new isoform variants by elongating the ends of known transcripts; however this was not investigated in our analysis.

### Ontology and pathway analyses of differentially spliced and expressed transcripts: candidate genes are mainly involved in cancer hallmarks and genetic disorders pathways

To identify biological pathways and functions enriched in our significant genes, we conducted gene ontology and pathway analyses with the set of genes that presented whole-gene expression changes and/or isoform differences between the tumor samples using the Ingenuity Pathways Analysis (IPA version 6.0) software package (Ingenuity Systems, Mountain View, CA). The IPA software compares its knowledge base - made up of all annotated genes in the genome - to the user gene data set uploaded into in the application. For each biological function or disease assigned, IPA uses the right-tailed Fisher's exact test to calculate a p-value determining the probability that the user dataset has more molecules associated with the biological function or disease than the reference set of molecules is due by random chance. Among the 2623 differentially expressed and/or spliced genes, 1224 have known molecular and cellular functions. This proportion includes genes previously reported to be involved in human diseases. We observed several differentially expressed and/or spliced genes that are implicated in cellular growth and proliferation, cellular death, tissue development, cell to cell signaling and interaction, cellular movement pathways, as well as in genetic disorders and cancers (Tables 2, 3). Some of the most interesting gene candidates in pathway interactions are described in detail below.

**Table 2:** Top height over-represented biological functions and diseases for genes with isoform variations or whole-transcript expression differences.

Function or disease^1^	# genes^2^	P-value^3^
Genetic disorder	567	5.45×10^−47^
Cancer	611	5.51×10^−43^
Cellular growth and proliferation	467	5.00×10^−33^
Cellular death	423	1.90×10^−29^
Tissue development	282	7.75×10^−28^
Cell-to-cell signaling and interaction	239	1.05×10^−21^
Cellular development	314	1.11×10^−20^
Cellular movement	254	5.14×10^−20^

The gene pathway analysis retrieved biological functions and/or diseases^1^ that were most significant to the candidate genes. For each function or disease, the number of significant genes^2^ involved is mentioned. The right-tailed Fischer's exact test p-value^3^ associated with a biological function or disease determines the likelihood that our set of significant genes has more molecules associated with the biological function or disease than the reference set of molecules is due by random chance. A gene could be involved in more than one function or disease.

**Table 3:** Examples of significant genes, in the gene pathway, having important implications in normal biological processes and cancer.

Gene symbol (RefSeq ID)^1^	Biological function^2^	Expression pattern^3^	pathological implication^4^
CD44 (NM_009851)	regulation of cell growth; cell adhesion; cell-matrix adhesion; cell-cell adhesion	High inclusion of three novel introns in 4T1.	Increases adhesion [23] and invasion [7] of breast cancer cells. Decreases cell death and apoptosis of tumor cells [24]. Increases cell death of normal cell [61]. Increase cell migration [6], movement [62] and binding [8] of tumor cells.
		High inclusion of exons 8, 10, 11 and 13 in 4T1	
PHB (NM_008831)	apoptosis, growth, proliferation, colony formation, cell cycle progression, migration, transmembrane potential, binding	Cassette exon: exon 4 over-expressed in 4T1	Negative regulator of cell proliferation and tumor suppressor [25], [26]
BTG1 (NM_007569)	proliferation, apoptosis, differentiation, growth	Differential 3′ UTR: the 3′ UTR region over-expressed in 4T1	Anti-proliferative gene that regulates cell growth and differentiation [63]
ITGB1 (NM_010578)	G1/S transition of mitotic cell cycle; cellular defense response; cell adhesion; positive regulation of cell proliferation; germ cell migration;	Intron inclusion between exons 9 and 10 in 4T1	Decreases cell death of tumor cells [64], [65]. Increases cell death of normal cells [66], [67]. Increases migration [68], cell adhesion [68] and cell binding [69] of tumor cells.
ANGPT2 (NM_007426)	angiogenesis; signal transduction; multicellular organismal development; cell differentiation	Alternative termination in 4T1	Increases cell death of normal cells [70].
HPRT1 (NM_013556)	purine nucleotide biosynthetic process; nucleoside metabolic process; protein homotetramerization	Cassette exon: exon 1 over-expressed in 168FARN and 4T07	Increases cell death of normal cells [71].
CCNT2 (NM_028399)	differentiation, apoptosis, proliferation	Intron inclusion between exons 6 and 7 in 168FARN and 4T07	Decreases apoptosis of tumor cells in colon cancer [72]
MAPK14 (NM_011951)	protein amino acid phosphorylation; cell motion; chemotaxis; response to stress; cell surface receptor linked signal transduction; protein kinase cascade;	Intron inclusion between exons 2 and 3 in 4T07	Increases cell death of normal cell [73], [74]. Increase developmental process of tumor cells [75].
SLK (NM_009289)	nucleotide-excision repair; protein amino acid phosphorylation; apoptosis	Cassette exon: exon 13 highly expressed in 4T1	Increases cell death of normal cells [76].

For each gene, the symbol and the RefSeq accession number^1^, the biological function^2^, the type of splicing event^3^ and the pathological implication^4^ are given^3^.

**Figure 3:**
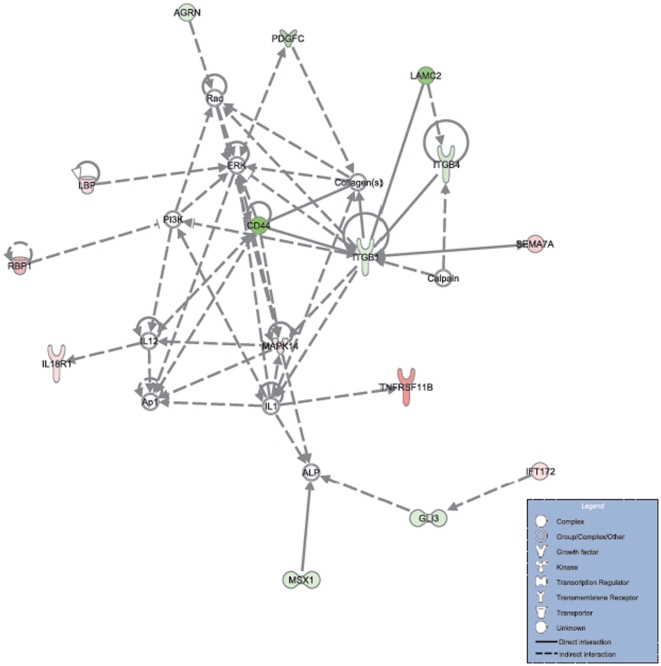
A network of molecular interactions containing differentially spliced or expressed genes between breast cancer tumors of varying metastatic phenotype. Over- or under- expressed genes in 4T1 compared to 168FARN and 4T07 are respectively indicated by a green or a red color of the gene-product icon. The over- or under-expression rate is proportional to the color intensity. Genes that are not colored are those that are not differentially expressed or spliced in our data. The top functions or diseases where the gene-product are involved are cancer, tissue development, cell-to-cell signaling and interaction.

The CD44 gene in normal biological conditions is involved in regulation of cell adhesion, proliferation and migration. It has been previously reported to present an inclusion of 10 internal variable exons (from exons 6 to 15) in mammary tumorigenesis [9]. CD44 is known to interact with MAPK1, a member of the Erk (extracellular signal-regulated kinase) complex, and also to a complex of collagen proteins (Figure 3). Collagens are the most abundant proteins in the extracellular matrix where it plays an essential function in the organization of cells. Many candidate genes, including several 4T1-overexpressed collagen subunit genes, interact with Erk which is a complex consisting of MAP kinase proteins and playing a role in cell division, growth and proliferation (Supplementary Figure S1). Erk phosphorylates many cytoplasmic and nuclear substrates required for the transcription of several genes to pass from the G1 stage to the S stage in the cellular division process [30]. There is evidence that in breast cancer, the inhibition of ERK enhances the anti-estrogenic treatment [31].

Another interesting example is CDH1, a tumor suppressor gene [32] from the cadherin superfamily that encodes an epithelial cell-cell adhesion protein. CDH1 acts on NF-κb, F-Actin and Mapk complexes (Supplementary Figure S2), all of them greatly implicated in cell-cell interactions, development and cell movement. This candidate gene encodes a protein that facilitates calcium-dependent homophilic interactions at cell-cell contact sites known as adherens junctions. Mutations in this gene are related to tumor cell growth and invasion in gastric, thyroid, colorectal and ovarian cancers [33], [34], [35]. The low expression level or the loss of E-Cadherin function is thought to contribute to cancer progression by increasing proliferation, invasion and metastasis [33], [34]. In 4T1 tumor cells, CDH1 was highly expressed compared to 168FARN or 4T07 cells (Additional file 1). This finding conflicts with the expected low CDH1-expression level in highly metastasis tumor. Given that primary tumor cells require adhesion within the vasculature to invade distant organs via the bloodstream [36], [37], [38], we hypothese that E-Cadherin could support tumor cell attachment to the vessel wall to spread to distant sites via the blood flow which is the main route by which primary tumor cells invade to distant sites. Besides CDH1, several other significant genes (such as LGALS7, ZFAND5, LSP1, MAPK14 and HSF1) interact with the NF-κB complex that is involved in autoimmune response, inflammation, cell proliferation and cell death by controlling the expression of genes implicated in these processes [39]. The supplementary Figure S2 shows a sub pathway highlighting some of these interactions. Misregulation of NF-κB has been associated with cancer, autoimmune diseases and inflammatory responses [40], [41]. Hence, the gene pathway analysis identified differentially spliced and/or expressed candidate genes during breast cancer metastasis that may have biological and pathological relevance.

However, there were many highly significant genes showing novel isoforms in our data that were not previously associated with cancer. Some examples are IFT172, ACSBG1, MED24, AGRN and CPXM2. The MED24 (mediator complex subunit 24) gene acts indirectly on VDR [42], a 4T1-upregulated gene in our data, which functions to inhibit p38 activity – a known mediator of tumor cell death in colon cancer [43]. Hence, the gene pathway investigation identified genes of which the functional relevance to pathologies is not currently known and that could potentially be linked to the process of breast cancer metastasis.

### qRT-PCR validation of selected candidate genes confirms the predicted alternative splicing events

In order to verify the presence of alternative splicing events that emerged from our analysis, we performed RT-PCR assays of ten selected candidate ASEs in MED24, SLC39A14, MFI2, SRRT, CD44, CLK1 and HNRNPH1, (see Additional files 1 and 2 for expression patterns of candidates). We amplified the differentially spliced regions in the various tumor samples by RT-PCR using primers pairs flanking and/or inside these regions (Supplementary Table S1). The expected PCR products were readily obtained for all candidate ASEs except for the SLC39A14 ASE and the SRRT- intron inclusion between exons 13 and 14. For these two cases, we obtained very little to no amplified product. To further quantify the products, we performed quantitative real time RT-PCR using tumor samples from 168FARN and 4T1 for six candidate ASEs that displayed positive qualitative RT-PCR results: MED24 (alternative start from exon 20 in 4T1), CD44 (from 5′ to 3′ ends, first retained intron in 4T1), CD44 (intron inclusion between exons 9 and 10 in 4T1) and SRRT (intron inclusion between exons 5 and 6 and CLK1 - intron inclusion between exons 7 and 8 in 4T1). The RT-PCR results across samples for the differentially spliced gene regions of MED24, CD44 and SRRT were in agreement with the corresponding expression data from the exon array (Supplementary Figures S3, S4, S5).

*MED24*: We quantified the amplification of four distinct genomic regions within the MED24 gene to assess the alternative start event. (1) To quantify the constitutive region of MED24 expressed in all three tumor samples (region from exons 1 to 19), we designed a primer pair spanning the region from exon 16 to exon 18. Since all the exons in the constitutive region were approximately equally expressed, the confirmation of some of them can be extended to the rest of the region. The region covered by this primer pair was amplified at roughly equal levels in both samples 168FARN and 4T1 (Supplementary Figure S3-A); that was expected since the exon array showed no expression variation between samples for this region. (2) We amplified the region spanning the breakpoint between the constitutive block and the alternative block (region from exons 20 to 26) of the transcript predominantly expressed in 4T1. The primers pair designed for this purpose covered the region from exons 19 to 20. The quantitative real time RT-PCR results showed that this region was roughly amplified at the same level in both 4T1 and 168FARN (Supplementary Figure S3-B) and therefore proved that there was an expression continuity between the constitutive block and the alternative in both 168FARN and 4T1. This observation led us to conclude that the whole transcript of MED24 is expressed in both 168FARN and 4T1. (3) We set a pair of primers (from exon 21 to exon 23) to measure the alternative region which was over-expressed in 4T1. The quantitative real time RT-PCR clearly confirmed that the alternative block is abundant in 4T1 comparatively to 168FARN (Supplementary Figure S3-C). (4) We wanted to exclude the hypothesis that the over-expression of the alternative block in 4T1 compared to 168FARN signified an alternative end of the transcript in 168FARN by the lack of the alternative block. We found in public sequence databases mRNA evidence (Genebank id: AK020269) that supports an early end of MED24 and fully maps to the constitutive block from our study. Therefore, we quantified the region corresponding to the end of this mRNA. We observed much product variance between and within replicate samples (Supplementary Figure S3-D), suggestive of very low expression of that region. Given that the results of this amplification assay were not convincing and that additionally this region had no probe coverage in the exon array, we had no evidence for an alternative end of MED24. Moreover, an alternative end in 168FARN would show a higher expression of the constitutive block than the alternative block, but the exon array showed that the constitutive block and the alternative block are expressed at the same level in sample 168FARN (Figure 1A, bottom panel). Altogether, the quantitative real time RT-PCR results for MED24 reproduced the corresponding expression data from the exon array and gave strong evidence that the whole transcript was expressed in all the samples, but the alternative block was enriched in 4T1 compared to 168FARN and 4T07 and is expressed as an additional independent transcript, most likely using a novel, unnanotated promoter.

*CD44*: We measured two of the three novel CD44 intron inclusions in the quantitative real time RT-PCR experiment (the first intron retained in 4T1 and the intron inclusion between exons 9 and 10 in 4T1). The qRT-PCR products obtained were consistent with our prediction (Supplementary Figure S4). Additionally, we successfully sequenced the RT-PCR products of these three introns, mapped the sequence to the mouse genome using BLAT [44], and displayed it in the UCSC Genome Browser (Figure 1C). The novel identified intron inclusions do not overlap any coding mRNA or EST sequences and public sequence databases have extremely weak unspliced EST evidences showing splicing events involving these regions, but no observation for the introns themselves. Therefore they are novel splicing events and elongate the list of the ten CD44 cancer-specific variable exons.

## Discussion

In this work, we used a splicing-sensitive microarray technology to investigate changes in pre-mRNA splicing that are associated to the formation of macroscopic metastases. Thus we focussed on tumor cells that could escape the primary site but fail to form macroscopic metastases (4T07, 168FARN) and those that can leave the primary site and form growing metastases (4T1). Using several stringent statistical selection criteria and filtering steps on probe sets (exons) and meta-probe sets (transcripts), we obtained a confident set of 2623 candidate genes that undergo isoform or whole gene expression variations specific to metastatic characteristics. Due to the inherent difficulty of unambiguously interpreting statistically significant expression changes in Exon Array data [45], only 7.7% of gene variations were classified into the gene-expression change or known splicing change categories. The remaining changes (92.3%) were difficult to interpret and may reflect the complexity of gene expression variation in cancer. For example, a single isoform may arise from multiple ASEs, which makes interpretation of the expression pattern of such a transcript very difficult to interpret. Isoform differences occurring in cancer cells are not always crystalline and explained by standard known changes. Although the Exon Array is a powerful tool, some ASEs may be missed or misinterpreted by Exon Array. For example, a fundamental limitation of Exon Array, or any other approach to measure gene expressions changes, is the reality that splicing events can introduce aberrations in the transcript such as premature stop codons. If these transcripts are degraded at a high rate by the RNA surveillance mechanisms (nonsense-mediated decay), their expression levels may be detectable only partially or not at all since they are unstable in the cell. Hence, the analysis presented here uncovers only a part of the transcriptional and post-transcriptional aberrations that distinguish mammary tumors of differing metastatic phenotype. As a first step to understanding the biological significance of alternatively spliced genes, we carried out a global gene pathway analysis. We found that nearly half of the significant genes have previously been reported to be involved in cellular functions for which deregulations are related to cancer. We also identified strongly significant genes that have not previously linked to pathogenesis. It is tempting to speculate that these genes may represent novel oncogenes or specific modifiers of breast cancer metastasis. Importantly, we have validated some predicted ASEs by qRT-PCR and a high validation rate (80%, eight validated events out of ten) demonstrated the efficiency of the exon array and our data mining approach to identify gene regions that are significantly enriched in one tumor type relative to another.

Additionally we compared our outcome with a recent study (Dutertre *et al*, 2010 [46]) that used tumors derived from the same cell lines (168FARN, 4T07 and 4T1), with the addition of the non-metastatic 67NR cell line, which forms primary tumors but fail to form distant metastases. This study assayed the tumor RNA expression profiles on the exon array platform to implement a prognostic classifier for breast cancer clinical outcome based on splicing variants in breast cancer metastasis. They obtained a total of 679 differentially spliced genes, including 209 genes with at least one annotated alternative exon. Importantly, 62 of these 209 genes had significant evidence for alternative splicing in our study. Of these, 20 showed at least one consistent ASE between the two studies while the remaining had different predicted events (Table S2). Some of the consistent genes have been previously described to be strongly related to cancer. An example is BCL2L11 that encodes a protein belonging to the BCL-2 family, which is composed of several pro- or anti–apoptotic regulators [47]. BCL2L11, which plays a crucial role in apoptosis initiation, is frequently mutated in various human tumors leading to the loss of its function [48], [49]. In both independent studies, the analyses showed that intron 2 is differentially expressed between tumor samples. This intron disrupts a BCL-X interacting domain in the protein product. Another common example is SPINT2, a protein that is over-expressed in pancreatic cancer and participates in tumor cell invasion and metastasis [50]. Both studies found SPINT2-exon 4 differentially expressed, a part of a Kunitz/Bovine pancreatic trypsin inhibitor domain in the protein. The confident set of common candidate genes between the two independent studies strongly suggests that they may have essential roles in breast cancer metastasis.

We used the Dice similarity coefficient [51] to evaluate the closeness of our results with those (209 genes) from Dutertre's study. We obtained a Dice coefficient of 0.044 (2×62/209+2623) - with measure ranges from 0 (22×0/209+2623) to 0.148 (2×209/209+2623) in this case – indicating an overlap of 30% (0.044/0.148×100) between the two outcomes. The estimation of statistical significance revealed a significant overlap between the two studies (One-tailed Chi-square test p-value = 1.17×10^−2^), providing further evidence that many of the significant genes that are common to both studies may be implicated in breast cancer metastasis – as they were detected by independent groups, using slightly different experimental approaches. It should be noted that larger outcome size in our study, as compared to Dutertre's study, resulted from our additional profiling of non-annotated gene regions that were not considered in Dutertre's study and that revealed several intronic regions which were differentially expressed between tumors with different metastatic potential. However the candidate genes shared by both studies but with different predicted outcome led us to hypothesize that one of the confounding factors that affect tumor behaviors across labs may be the age, the volume, the growth rate of the primary tumors at the time of removal, the experiment conditions, the genetic instability of the cell lines, the injection site and the computational analysis for data mining. These factors alone or combined could influence the expression profile of gene.

In conclusion, the connection between breast cancer and gene splicing alteration is becoming increasingly compelling based both on prior published data and our present work. Our data suggests that, during breast cancer metastasis, numerous genes display expression variations and/or splicing defects whose encoded protein products could disturb normal biological processes. Compared to other approaches based on DNA microarrays that interrogate single whole genes, studying the transcriptome at the exon level provides an accurate detailed knowledge about the variations occurring within the genes and this could lead to improved and more specific diagnostics or therapies. Moreover, we demonstrate that non-coding gene regions, which are not normally expressed can be differentially incorporated into transcripts within breast cancers of differing metastatic phenotype. We also observe that the proportion of intron inclusion was higher in breast cancer cells with the greatest metastatic potential. Thus, we suggest that aggressively metastatic breast cancer cells are prone to splicing misregulation of non-coding gene regions. By establishing which genes actively participate during different stages of metastasis, it is possible that metastasis-specific isoforms may emerge as breast cancer biomarkers [52]. Indeed, identified novel isoforms or known isoforms that are not currently implicated in any disease can be investigated as novel therapeutic targets [53], [54] in mammary tumors. For instance, a therapeutic small interfering RNA (siRNA) [55], [56] approach can be used to specifically target and silence a gene isoform that disrupts a normal biological process critical to breast cancer. Further functional characterizations of gene regions alternatively spliced have the potential to improve the understanding of the complicated biological processes connecting isoform variations and the metastasis machinery.

## Materials and Methods

### Cell Culture

The 4T1 murine mammary carcinoma cell line was obtained from the American Type Culture Collection. Non-metastatic 168FRNA and 4T07 were kindly provided by Dr. Fred Miller (Barbara Ann Karmanos Cancer Institute, Detroit, MI). All cell lines were grown in DMEM supplemented with 10% fetal bovine serum, 10 mmol/L HEPES, 1 mmol/L sodium pyruvate, 1.5 g/L sodium bicarbonate, penicillin/streptomycin, and fungizone.

### Mammary gland injection

Female BALB/c mice (4–6 weeks) were purchased from Charles River Laboratories. The mice were housed in facilities managed by the McGill University Animal Resources Centre, and all animal experiments were conducted under a McGill University–approved Animal Use Protocol in accordance with guidelines established by the Canadian Council on Animal Care. Four mice were injected (1×10^−5^ cells) with 168FARN, five mice with 4T07 and four mice with 4T01. Tumor volumes were calculated using the following formula: *πLW^2^/6*, where *L* is the length and *W* is the width of the tumor. Tumors were surgically removed, using a cautery unit, once they reached a volume between 100 and 125 mm3. Tumor tissues were frozen to avoid RNA degradation.

### RNA extraction and microarray hybridization

Tumor tissues were disrupted in a specific buffer, homogenized, and then RNA was purified using Rneasy Mini kit (Qiagen) following the manufacturer's instructions. RNA 6000 Nano Chips with the Agilent 2100 Bioanalyser (Agilent) were subsequently used, following the manufacturer's instructions, to verify the RNA quality, integrity and the lack of gDNA contamination. Tumors were hybridized independently at the functional genomics facility of McGill University and Genome Quebec Innovation Centre (Montreal, Quebec, Canada). Biotin-labeled target for the microarray experiment were prepared using 1µg of total RNA. We subjected the RNA to a ribosomal RNA removal process with the Ribo/Minus Human/Mouse Transcriptome Isolation kit (Invitrogen). cDNA was synthesized using the GeneChip® WT (Whole Transcript) Sense Target Labeling and Control Reagents kit as described by the manufacturer (Affymetrix). Then, the sense cDNA was fragmented by UDG (uracil DNA glycosylase) and APE 1 (apurinic/apyrimidic endonuclease 1) and biotin-labeled with TdT (terminal deoxynucleotidyl transferase) using the GeneChip® WT Terminal labeling kit (Affymetrix, Santa Clara, USA). Hybridization was performed using 5 micrograms of biotinylated target, which was incubated with the GeneChip® Mouse Exon 1.0 ST array (Affymetrix) at 45°C for 16–20 hours. Subsequently to hybridization, non-specifically bound material was removed by washing and detection of specifically bound target was performed using the GeneChip® Hybridization, Wash and Stain kit, and the GeneChip® Fluidics Station 450 (Affymetrix). The arrays were scanned using the GeneChip® Scanner 3000 7G (Affymetrix) and raw data was extracted from the scanned images and analyzed with the Affymetrix Power Tools software package (Affymetrix). The microarray data has been deposited in the Gene Expression Omnibus Database (accession: GSE21994).

### Data pre-processing and analysis

**Signal estimation.** Signal estimates were derived from the CEL files of the 13 arrays. The Affymetrix Power Tools software package (Affymetrix) was used to quantile normalize the probe fluorescence intensities and to summarize the probe set (representing exon expression) and meta-probe set (representing gene expression) intensities using a probe logarithmic intensity error model (PLIER [57]) for probe set and ITER-PLIER for meta-probe set. Presence or absence of probe set expression was determined by the Detection Above BackGround (DABG) statistics. For the probe set-level analysis we used the full set of probe sets from the Exon Array including core and non-core probe sets.

**Filtering signal data.** The filtering steps and parameters described in this paragraph come from the Affymetrix technical note for the identification of ASE using the exon array [58]. One outlier biological replicates from 4T07 which didn't cluster with the replicates within the tumor type it belonged to, was identified and removed following the Principal Component Analysis (PCA). In order to be considered as expressed and included in the analysis each exon had to satisfy the following four criteria (Supplementary Figure S6): (1) the exon is called as Present in at least 50% of the sample replicates of at least one tumor type. An exon is called as “present” if its probe set DABG p-value is less than 0.05; (2) the probe set must have a low cross-hybridization potential (equal to 1) to discard false positives. The signal intensities of probe sets having a high cross-hybridization potential may come from a different gene sequence; (3) the probe set must have a gene-level normalized intensity lower than 5 (very large gene-level normalized intensity may also implicate cross-hybridization to other genomic sequences); (4) the probe set must have a gene-level normalized intensity greater than 0.20 (very low gene-level normalized intensity probe sets were removed to discard features that may have non-linear signal response.) For each gene containing the previously filtered exons, two filtering criteria were used: (1) the gene had at least 50% of core exons called as “present” in at least 50% of sample replicates in at least two tumor types; (2) the ITER-PLIER gene intensity is greater than a threshold of 30.

We performed two concurrent analyses: AS analysis with the probe set intensities and AS analysis with the gene-level normalized intensities. There is no optimal method to analyze isoform level data, and the relative merits of each approach are described in some detail by Bemmo *et al.* [45]. For each analysis, a one-way ANOVA-test was done on probe set scores to retrieve probe sets that have a statistically significant change of expression between tumor groups. We selected probe sets having an ANOVA P-value lower than the P-value threshold (6.36×10^−4^ for probe set intensity analysis and 7.10×10^−4^ for SI analysis) established by the Benjamini-Hochberg FDR (False discovery rate) correction [59] at a 0.05 level. 168FARN and 4T07 were compared against 4T1 by pairwise Student's t-tests on probe sets scores. Logarithmic fold-changes were computed between groups (168FARN/4T1 and 4T07/4T1). The genes expression intensities of meta-probe sets were analyzed by the same way as probe sets. The statistical significance of a whole gene expression was determined by an FDR P-value threshold of 8.46×10^−3^ computed from Anova-test P-values. Since the SI analysis performs best when a gene has a large number of constitutive exons comparatively to alternative exons, we restricted the SI analysis to genes whose overall gene expression does not change. Fold-changes and P-values of exons within each gene have been uploaded and visualized in the UCSC Genome Browser environment.

The visualization enabled us to classify ASEs (Supplementary Figure S7). We examined the exon expression fold-changes within the gene: if the whole gene expression changed (P<8.46×10^−3^), we categorized it as a gene expression change and determined the possibly additional ASEs within the gene. If the whole gene expression didn't change (P≥8.46×10^−3^), we looked at the exon-level: if an exon expression within the gene changed (P<6.36×10^−4^ for probe set intensity analysis or P<7.10×10^−4^ for SI analysis), we categorized the ASE. Subsequently, we performed a gene pathway analysis of significant genes with the Ingenuity Pathways Analysis (IPA) software, version 6.0 (Ingenuity Systems, Mountain View, CA).

### Validation of alternative splicing events by RT-PCR and qRT-PCR

We used a different RNA batch of the same tumors for the PCR experiments. Total RNA was treated with 4U of DNAase I (Ambion) for 30 minutes to remove any remaining genomic DNA. First strand complementary DNA was synthesized using random hexamers (Invitrogen) and Superscript II reverse transcriptase (Invitrogen). All the candidate probe sets were internal and possessing flanking exons in known RefSeq and mRNA isoforms. We designed locus specific primers within the adjacent flanking exons for the RT-PCR reaction (supplementary Table S1) by using the Primer3 v. 0.4.0 software [60]. For MED24 gene for which we predicted an alternative start in 4T1, additional primer pairs were designed to amplify products corresponding to the adjacent probe sets and which were not statistically significant. Approximately 20 ng of total cDNA was then amplified by PCR using Hot Start Taq Polymerase (Qiagen, Mississauga, Canada) with an activation step at 95°C (15 min) followed by 35 cycles at 95°C (30 s), 58°C (30 s) and 72°C (40 s) and a final extension step at 72°C (5 min). Amplicons were visualized by electrophoresis on a 2.5% agarose gel.

We performed a quantitative real time PCR in 168FARN and 4T1 for the primers pairs for which the qualitative RT-PCR product were positively conclusive. Real-time analysis was carried out using Power SYBR Green PCR Mix (Applied Biosystems) following the manufacturer's instructions on an ABI 7900 HT (Applied Biosystems) instrument. The reaction was set up in 10ul final volume applying the following conditions: 8ng of total cDNA and 0.32 uM of gene specific primers, and cycling, 95C (15min), 95C (20s), 58C (30s), 72C (45s) for 40 cycles. Relative quantification of each amplicon was evaluated in RNA from the tumor samples in biological duplicates each in technical triplicates. For each amplicon, including the mouse GAPDH used as endogenous control, a standard curve was established using dilution series of a mix of cDNA samples with known total cDNA concentration in order to determine if the amplification reactions had the same PCR efficiency. The cycle threshold (Ct) values for each replicate were transformed to relative concentration using the estimated standard curve function (SDS 2.1, Applied Biosystems) and normalized based on GAPDH real-time data from the same samples to account for well-to-well variability. Furthermore, CD44 RT-PCR products for two intron inclusion events were purified and cloned for sequencing.

## Supporting Information

Figure S1 A network of molecular interactions containing differentially spliced or expressed genes between breast cancer tumors of varying metastatic phenotype. Over- or under- expressed genes in 4T1 compared to 168FARN and 4T07-derived breast cancers are indicated by a green or a red color of the gene-product icon, respectively. The degree of over- or under-expression is proportional to the color intensity. Genes that are not colored are those that are not differentially expressed or spliced in our data. The top biological functions/processes or diseases where the gene-products are involved are cancer, cell cycle and cell death.
(0.15 MB PDF)

Figure S2 A network of molecular interactions containing differentially spliced or expressed genes between breast cancer tumors of varying metastatic phenotype. Over- or under- expressed genes in 4T1 compared to 168FARN and 4T07-derived breast tumors are indicated by a green or a red color of the gene-product icon, respectively. The degree of over- or under-expression is proportional to the color intensity. Genes that are not colored are those that are not differentially expressed or spliced in our data. The top biological functions/processes or diseases where the gene-products are involved are cancer, cell morphology, cell-to-cell signaling and interaction.
(0.18 MB PDF)

Figure S3 Quantitative RT-PCR validation of MED24 alternative start. In each panel, tumor samples are plotted on the x-axis and real time RT-PCR GAPDH-normalized quantities on the Y axis. We plotted the real time RT-PCR quantities for (A) the constitutive block (region from exons 1 to 19), (B) the region spanning the break point between the constitutive block and the alternative block (from exon 16 to exon 18), (C) the alternative block (region from exons 20 to 26) and (D) the end of the constitutive block (region from exons 21 to 23). We evaluated each tumor sample in two biological replicates each ran in three technical replicates.
(0.21 MB PDF)

Figure S4 Quantitative RT-PCR validation of CD44 4T1-intron retentions. In each panel, tumor samples are plotted on the x-axis and real time RT-PCR GAPDH-normalized quantities on the Y axis. We plotted the real time RT-PCR quantities for the first retained intron between exons 5 and 6 (A) and the intron inclusion between exons 9 and 10 (B). We evaluated each tumor sample in two biological replicates, each ran in three technical replicates.
(0.14 MB PDF)

Figure S5 Quantitative RT-PCR validation of SRRT 168FARN-intron retention between exons 5 and 6. The tumor samples are plotted on the x-axis and real time RT-PCR GAPDH-normalized quantities on the Y axis. We conducted qRT-PCR in two biological replicates (two independent tumors), each performed in triplicate.
(0.09 MB PDF)

Figure S6Workflow summary of the probe set and meta-probe set signal filtering steps. Signal estimates were derived from the CEL files. The probe logarithmic intensity error (PLIER) model was used to summarize the probe set (representing exon expression) intensities while the ITER-PLIER was used for meta-probe set (representing gene expression) intensities. The Presence or absence of probe set was determined by the Detection Above background (DABG) p-value. In order to be considered as expressed and included in the analysis each exon had to satisfy the following three criteria: (1) expressed in at least one sample; (2) the cross-hybridization potential equals to 1; (3) the gene-level normalized intensity greater than 0.2 and lower than 5. For each gene containing the previously filtered exons, two filtering criteria were used: (1) the gene is expressed in at least two samples; (2) the gene intensity is greater than a threshold of 30. We simultaneously performed either a one-way ANOVA test on probe set intensities or a one-way ANOVA test on meta-probe set intensities. Subsequent to ANOVA tests, we applied a 0.05-level FDR (False discovery rate) correction to determine the p-value threshold to identify significant exons (P<6.36*10
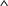
−4 for the probe set expression analysis; P<7.10*10
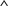
−4 for the SI analysis) and the significance of whole gene expression (P<8.46*10
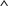
−3: over-expressed in a sample). Differentially expressed exons between paired samples were located by performing pairwise T-test comparisons (168FARN and 4T07 against 4T1). The log2 transformed expression fold-changes (4T07/4T1 and 168FARN/4T1) between paired sample comparisons were also computed.(0.07 MB PDF)

Figure S7 Workflow summary of the gene visualisation and the manual curation for gene variation pattern classifications. The probe set p-values and the probe sets fold-changes of T-test pairwise comparisons are visualised in the context of gene belongings to categorize transcripts variations.
(0.06 MB PDF)

Table S1 List of primers for validation. List of primers used in the qualitative and quantitative RT-PCR validation. Probe sets in blue are the target amplification probe sets whereas probe sets in black are flanking probset spanned or containing the primers.
(0.01 MB PDF)

Table S2 Common significant differently expressed exons between our study and Dutertre's study. The gene symbol, the gene name, the differentially spliced regions in our study and the differentially spliced regions in Dutertre's study are given. The records highlighted in bold represent common genes with at least one common predicted spliced regions.
(0.03 MB PDF)

File S1 UCSC browser links illustrating probe set level expression differences (fold-change and p-values) for the top 143 isoforms differentially expressed between the samples, obtained from the probe set level analysis.
(0.15 MB PDF)

File S2 UCSC browser links illustrating the probe set level expression differences (fold-change and p-values) as well as the normalized (SI) differences for the top 60 isoforms differentially expressed between the samples, obtained from the Splicing Index analysis.
(0.10 MB PDF)
