# Supplementary figures and images for: Exon-Level Transcriptome Profiling in Murine Breast Cancer Reveals Splicing Changes Specific to Tumors with Different Metastatic Abilities
Source: PLoS One. 2010 Aug 6;5(8):e11981. doi: 10.1371/journal.pone.0011981 (PMC2917353; doi:10.1371/journal.pone.0011981)

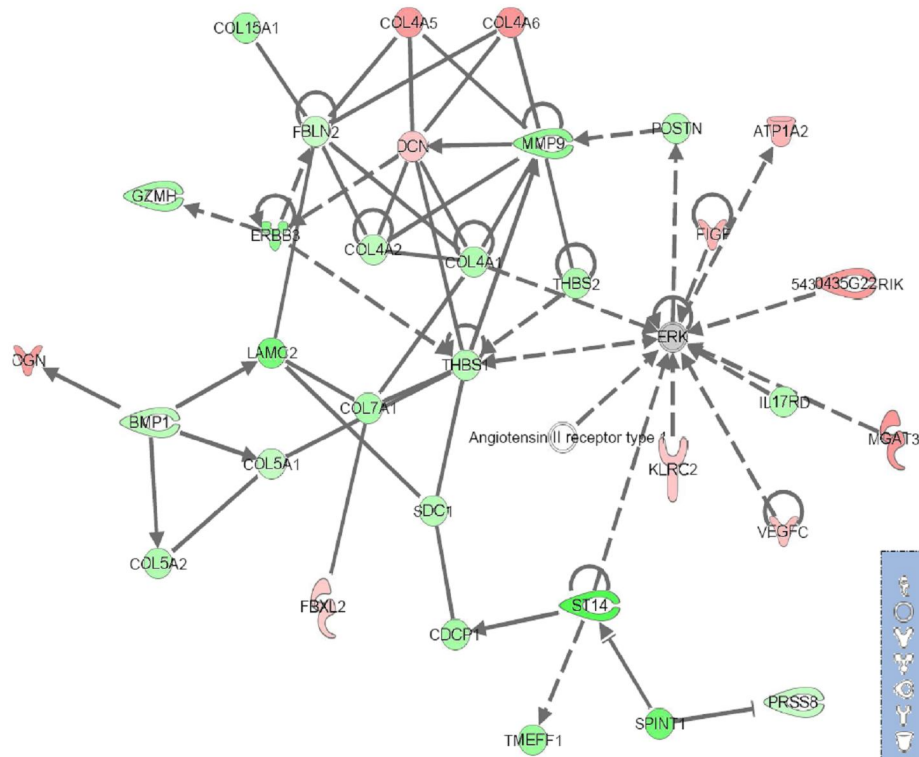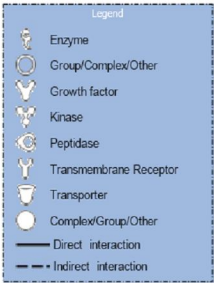

Supplement: Figure S1 — A network of molecular interactions containing differentially spliced or expressed genes between breast cancer tumors of varying metastatic phenotype. Over- or under- expressed genes in 4T1 compared to 168FARN and 4T07-derived breast cancers are indicated by a green or a red color of the gene-product icon, respectively. The degree of over- or under-expression is proportional to the color intensity. Genes that are not colored are those that are not differentially expressed or spliced in our data. The top biological functions/processes or diseases where the gene-products are involved are cancer, cell cycle and cell death. (0.15 MB PDF) [file pone.0011981.s001.pdf]

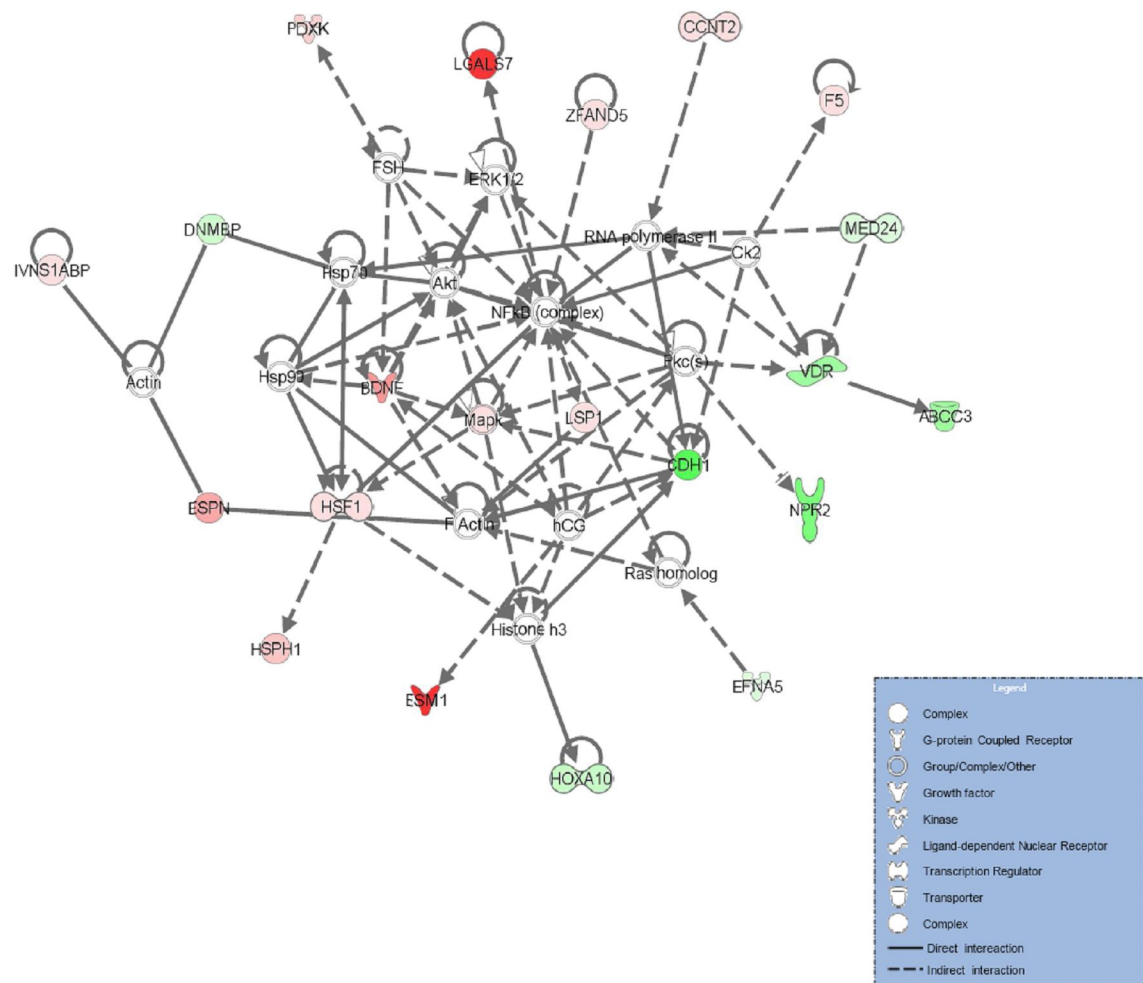

Supplement: Figure S2 — A network of molecular interactions containing differentially spliced or expressed genes between breast cancer tumors of varying metastatic phenotype. Over- or under- expressed genes in 4T1 compared to 168FARN and 4T07-derived breast tumors are indicated by a green or a red color of the gene-product icon, respectively. The degree of over- or under-expression is proportional to the color intensity. Genes that are not colored are those that are not differentially expressed or spliced in our data. The top biological functions/processes or diseases where the gene-products are involved are cancer, cell morphology, cell-to-cell signaling and interaction. (0.18 MB PDF) [file pone.0011981.s002.pdf]

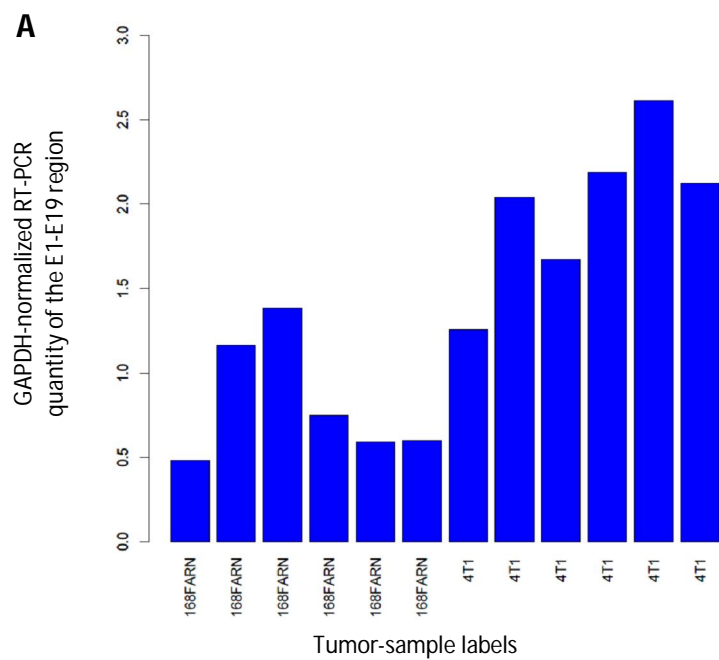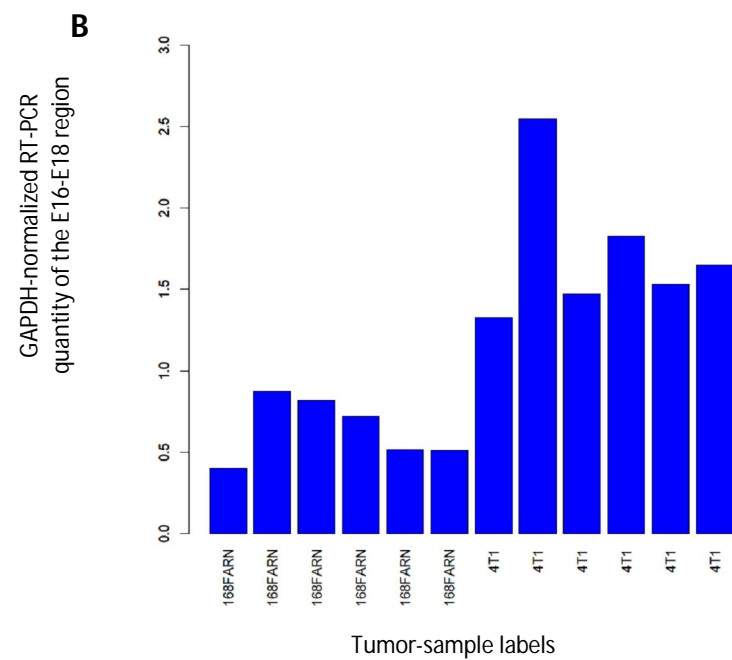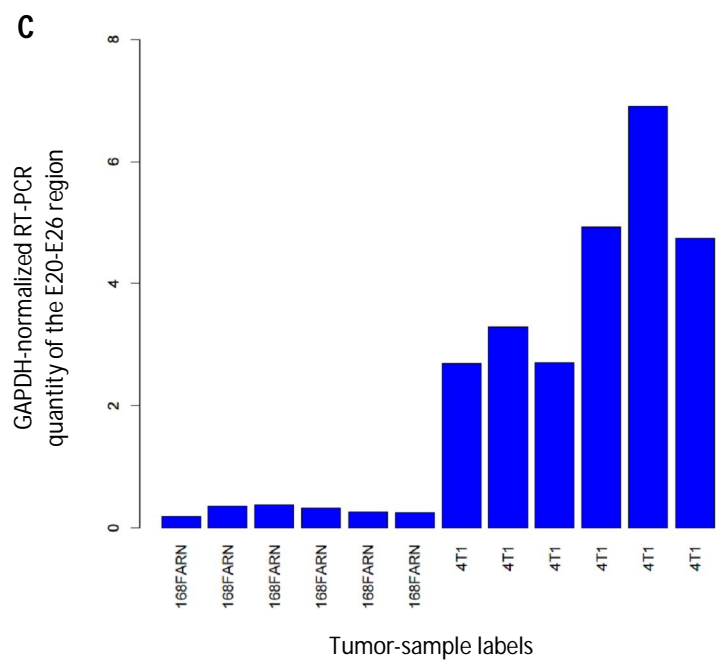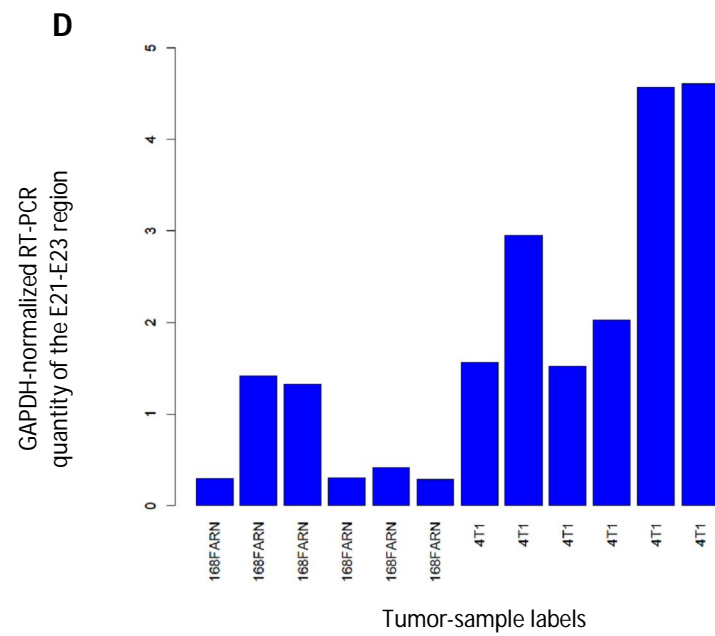

Supplement: Figure S3 — Quantitative RT-PCR validation of MED24 alternative start. In each panel, tumor samples are plotted on the x-axis and real time RT-PCR GAPDH-normalized quantities on the Y axis. We plotted the real time RT-PCR quantities for (A) the constitutive block (region from exons 1 to 19), (B) the region spanning the break point between the constitutive block and the alternative block (from exon 16 to exon 18), (C) the alternative block (region from exons 20 to 26) and (D) the end of the constitutive block (region from exons 21 to 23). We evaluated each tumor sample in two biological replicates each ran in three technical replicates. (0.21 MB PDF) [file pone.0011981.s003.pdf]

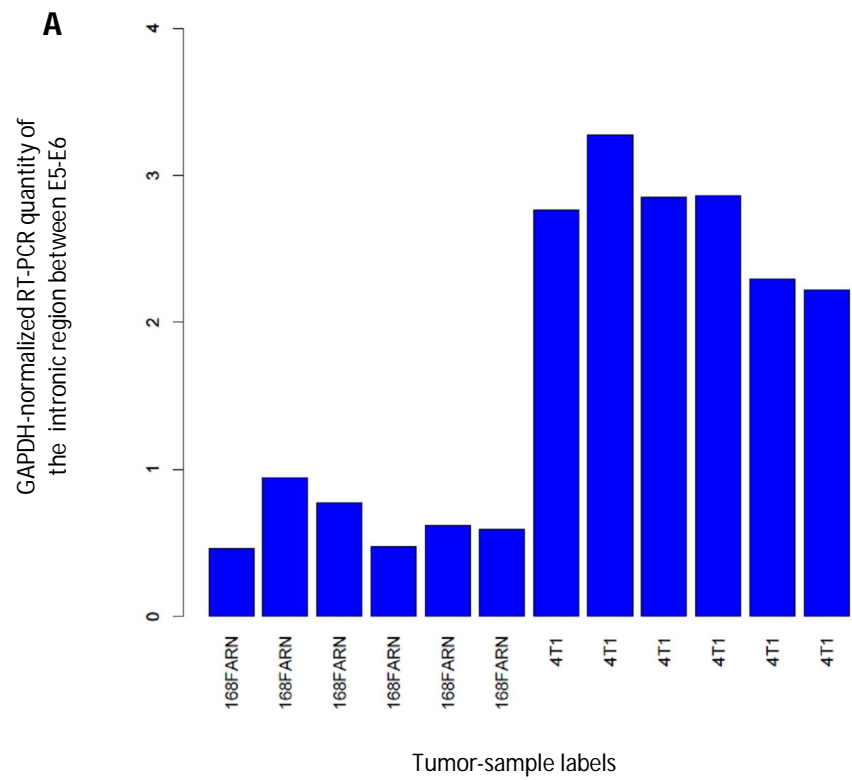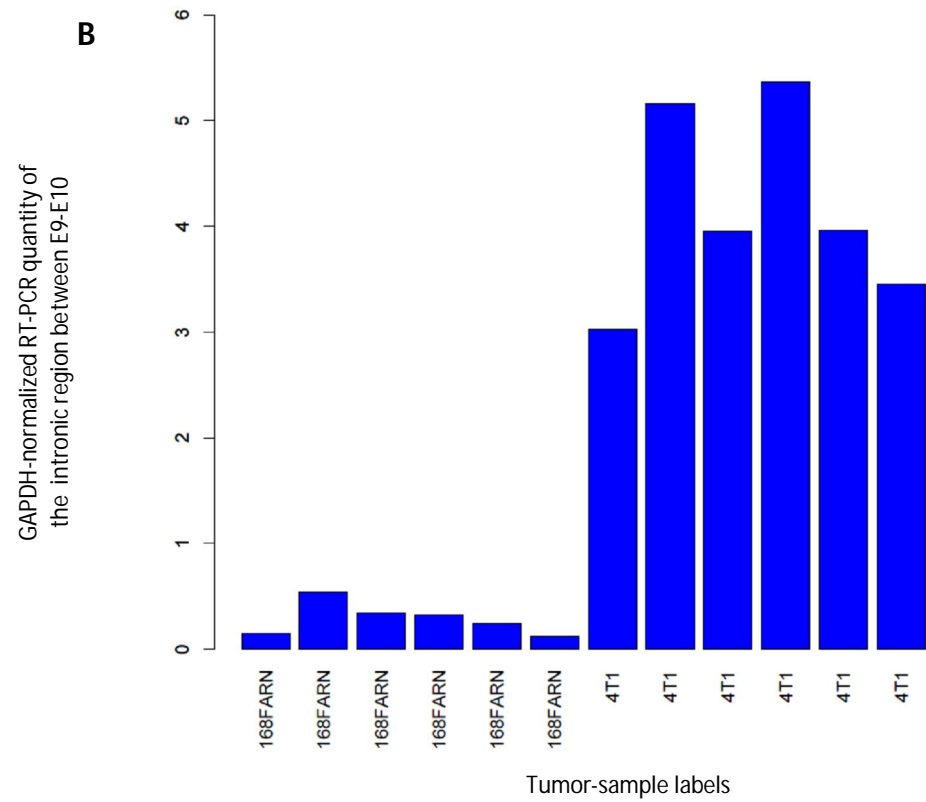

Supplement: Figure S4 — Quantitative RT-PCR validation of CD44 4T1-intron retentions. In each panel, tumor samples are plotted on the x-axis and real time RT-PCR GAPDH-normalized quantities on the Y axis. We plotted the real time RT-PCR quantities for the first retained intron between exons 5 and 6 (A) and the intron inclusion between exons 9 and 10 (B). We evaluated each tumor sample in two biological replicates, each ran in three technical replicates. (0.14 MB PDF) [file pone.0011981.s004.pdf]

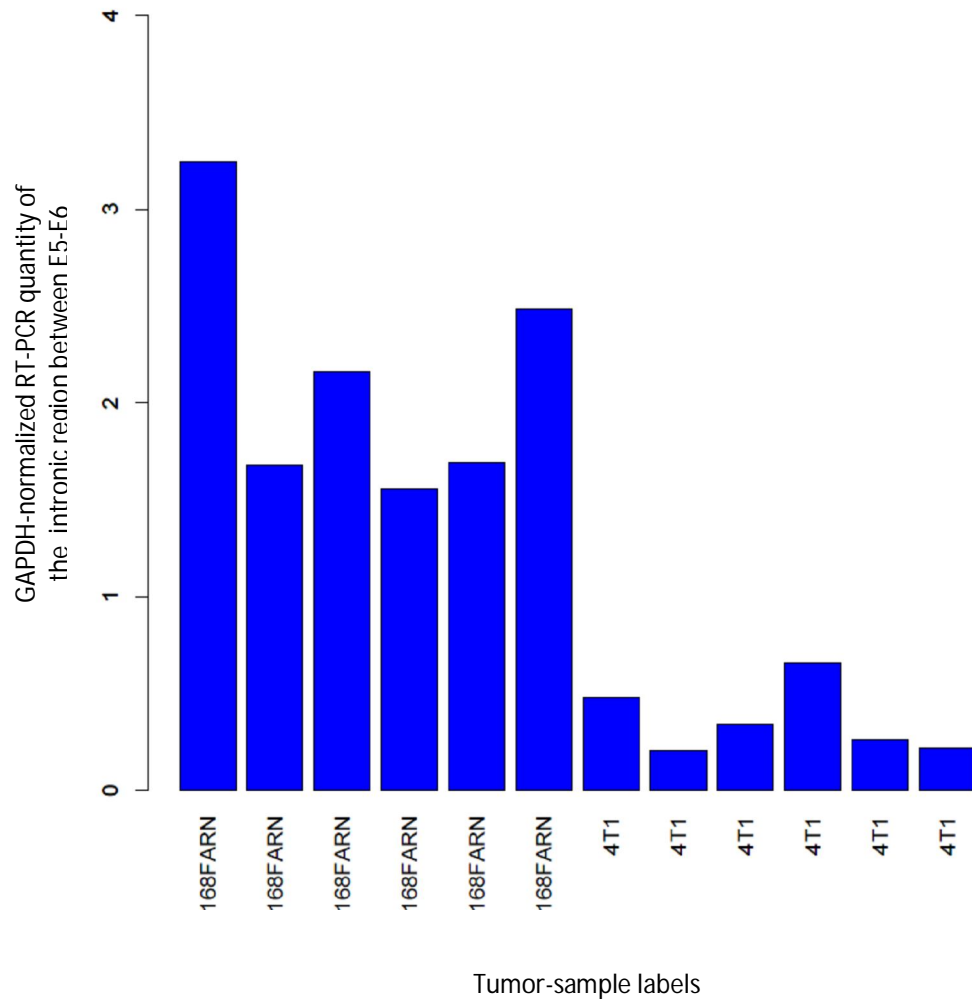

Supplement: Figure S5 — Quantitative RT-PCR validation of SRRT 168FARN-intron retention between exons 5 and 6. The tumor samples are plotted on the x-axis and real time RT-PCR GAPDH-normalized quantities on the Y axis. We conducted qRT-PCR in two biological replicates (two independent tumors), each performed in triplicate. (0.09 MB PDF) [file pone.0011981.s005.pdf]

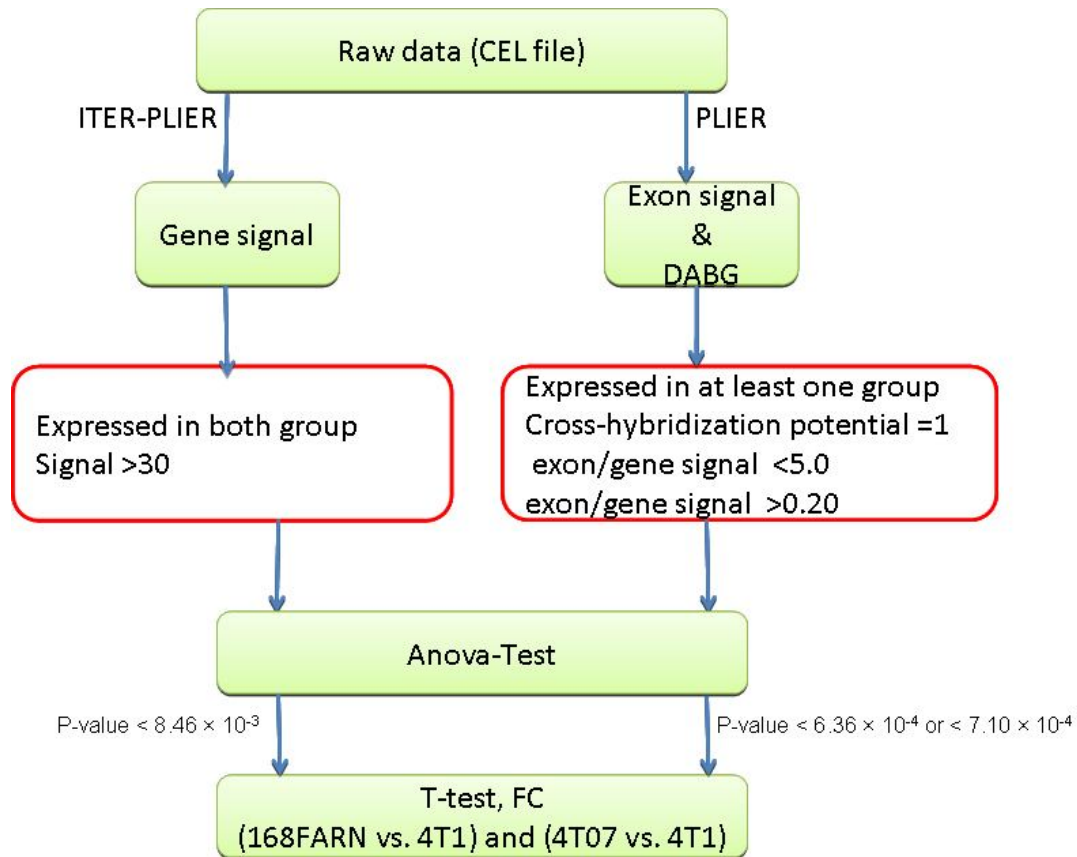

Supplement: Figure S6 — Workflow summary of the probe set and meta-probe set signal filtering steps. Signal estimates were derived from the CEL files. The probe logarithmic intensity error (PLIER) model was used to summarize the probe set (representing exon expression) intensities while the ITER-PLIER was used for meta-probe set (representing gene expression) intensities. The Presence or absence of probe set was determined by the Detection Above background (DABG) p-value. In order to be considered as expressed and included in the analysis each exon had to satisfy the following three criteria: (1) expressed in at least one sample; (2) the cross-hybridization potential equals to 1; (3) the gene-level normalized intensity greater than 0.2 and lower than 5. For each gene containing the previously filtered exons, two filtering criteria were used: (1) the gene is expressed in at least two samples; (2) the gene intensity is greater than a threshold of 30. We simultaneously performed either a one-way ANOVA test on probe set intensities or a one-way ANOVA test on meta-probe set intensities. Subsequent to ANOVA tests, we applied a 0.05-level FDR (False discovery rate) correction to determine the p-value threshold to identify significant exons (P<6.36*10−4 for the probe set expression analysis; P<7.10*10−4 for the SI analysis) and the significance of whole gene expression (P<8.46*10−3: over-expressed in a sample). Differentially expressed exons between paired samples were located by performing pairwise T-test comparisons (168FARN and 4T07 against 4T1). The log2 transformed expression fold-changes (4T07/4T1 and 168FARN/4T1) between paired sample comparisons were also computed. (0.07 MB PDF) [file pone.0011981.s006.pdf]

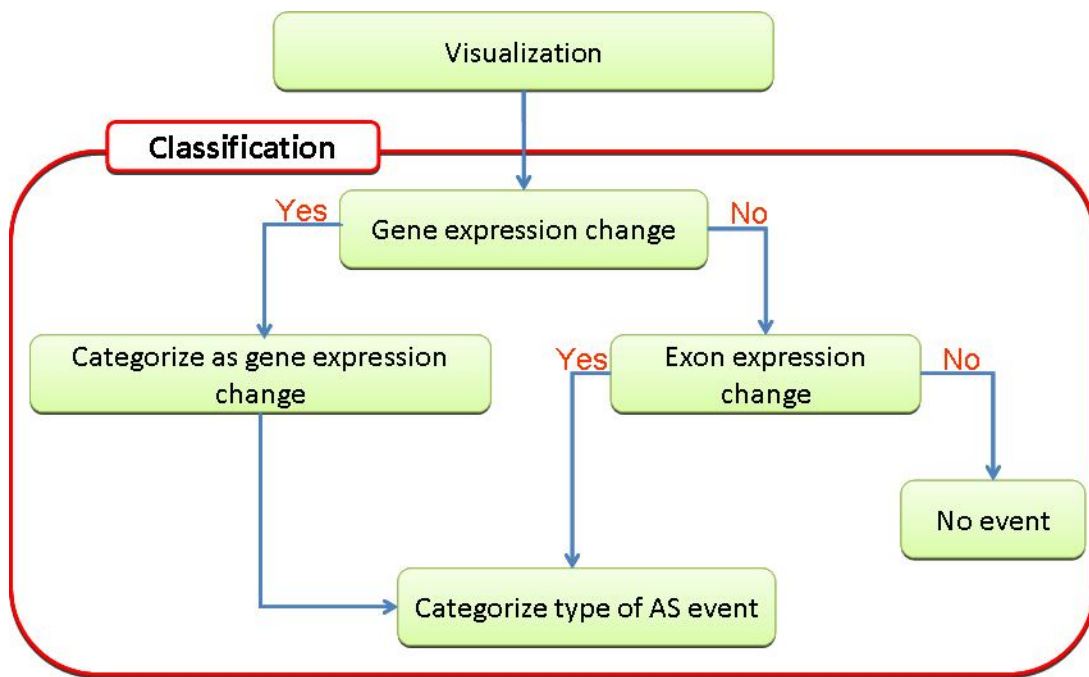

Supplement: Figure S7 — Workflow summary of the gene visualisation and the manual curation for gene variation pattern classifications. The probe set p-values and the probe sets fold-changes of T-test pairwise comparisons are visualised in the context of gene belongings to categorize transcripts variations. (0.06 MB PDF) [file pone.0011981.s007.pdf]
