# Supplementary material for: Exon-Level Transcriptome Profiling in Murine Breast Cancer Reveals Splicing Changes Specific to Tumors with Different Metastatic Abilities
Source: PLoS One. 2010 Aug 6;5(8):e11981. doi: 10.1371/journal.pone.0011981 (PMC2917353; doi:10.1371/journal.pone.0011981)
Supplement: Table S1 — List of primers for validation. List of primers used in the qualitative and quantitative RT-PCR validation. Probe sets in blue are the target amplification probe sets whereas probe sets in black are flanking probset spanned or containing the primers. (0.01 MB PDF) [file pone.0011981.s008.pdf]

Gene symbol	RefSeq accession number	Spanned Probe sets ID	Forward primer	Reverse primer
MED24	NM_011869	4461989	GCCACTGTACAGCGAGAACA	TGAAAAACGCCTGGAAGTCT
MED24	NM_011869	4461989, 5069415	GCCACTGTACAGCGAGAACA	GTTCCAGTAGGGCATCGTGT
MED24	NM_011869	4612541, 4511633, 4648201	ACAGCCTTATGGACCCTCCT	GGGACTTGACAGGATGTTGG
MED24	NM_011869	4655013, 5070661, 4461989	GCCCTGCTCAACAACTCTTC	GCCAGACTGCATACCTTTCC
SLC39A14	NM_144808	5797317, 5087649, 4872559, 4713911 4663713	ACAACTTGAGCGAGCGATCT	CACCACTGCAGACTTGGAGA
MFI2	NM_013900	4613686, 5508279, 5060504, 5284173	CGGTGAGCCAGTTCTTCAAT	CCATTGGGGCACAGTAGTTC
SRRT	NM_031405	5046135, 5382632, 4734614	GCAGATGCAGGACTTTTTCC	CATGAGGGACAGGAACACCT
SRRT	NM_031405	5046135, 5382632	GCAGATGCAGGACTTTTTCC	CAGTCAGAGGGCACATGAGA
SRRT	NM_031405	4830783, 4987145, 4577240	AACTGAGTCCCGGTGTGAAC	CTCTCTCCACGTTGATCTCG
CD44	NM_009851	5330587, 4423264, 4461784	CTCCAGACAACCACCAGGAT	TGTGGGGTCTCCTCTTCATC
CD44	NM_009851	4542928, 4622064	TCTTTATCCGGAGCACCTTG	CGGTGAAAAAGCCAGAGTTC
CD44	NM_009851	4622064, 5355815	TCCACTTCCCTCTCTGCACT	TGGGATAACCCAAGCCTACA
CD44	NM_009851	5355815, 5045988	GTGGAGGGCTGAGACACAGT	GGGCTCCTGAGTCTGAGTTG
CLK1	NM_001042634	5485817, 4418371, 5420215	AGTGGTTTGAGCATCGAGGT	TTTTGGGATTATAAGCCTCTGTG
CLK1	NM_001042634	4418371	GTGTGCCAGAGAGAGCATCA	TCACCAGAGAAGGTGTGAGG
CLK1	NM_001042634	4418371, 5420215	TGGCAACCCTCTGAGTTTTT	TTTTGGGATTATAAGCCTCTGTG
HNRNPH1	NM_021510	5351433, 4365208, 5558036	TTCACCACTCAACCCTGTGA	ATATCCGCTCATGCTGCTCT
GAPDH	NM_008084		AACTTTGGCATTGTGGAAGG	ACACATTGGGGGTAGGAACA
18sRNA	NM_011296		TGTGGTGTTGAGGAAAGCAG	TCCCATCCTTCACATCCTTC
