# Supplementary material for: Exon-Level Transcriptome Profiling in Murine Breast Cancer Reveals Splicing Changes Specific to Tumors with Different Metastatic Abilities
Source: PLoS One. 2010 Aug 6;5(8):e11981. doi: 10.1371/journal.pone.0011981 (PMC2917353; doi:10.1371/journal.pone.0011981)
Supplement: Table S2 — Common significant differently expressed exons between our study and Dutertre's study. The gene symbol, the gene name, the differentially spliced regions in our study and the differentially spliced regions in Dutertre's study are given. The records highlighted in bold represent common genes with at least one common predicted spliced regions. (0.03 MB PDF) [file pone.0011981.s009.pdf]

Gene symbol	Gene name	Differentially expressed Exons in our study	Differentially expressed exon in Dutertre et al study
**Scara5**	**scavenger receptor class A, member 5 (putative)**	**Exons 2-4, 6, 9, 10**	**Exon 10**
**Kirrel3**	**Kin of IRRE like 3 (Drosophila)**	**Exons 1, 3-14**<br><br>**Ten intron inclusions between exons 1 and 2**	**Exon 10**
**Cpxm2**	**carboxypeptidase X (M14 family), member 2**	**Exons 2-14**	**Exon 9**
**Masp1**	**mannan-binding lectin serine peptidase 1 (C4/C2 activating component**	**Exons 2-10**<br><br>**Two intron inclusions between exons 10 and 11**	**Exon 10**
**Dnm1**	**dynamin 1**	**Exons 1-16, 18, 19, 21**<br><br>**Two intron inclusions between exons 10 and 11**	**Exons 10, 11**
**Pdk1**	**pyruvate dehydrogenase kinase, isozyme 1**	**Exons 1-4, 6-11**<br><br>**One intron inclusion between exons 20 and 21**	**Exon 2**
Ece2	endothelin converting enzyme 2	Exons 4, 6-8, 11, 12, 15-17,	Exon 3
Dcn	decorin	Exons 1, 3-9	Intron 6
**Sned1**	**sushi, nidogen and EGF-like domains 1**	**Exons 2-31**<br><br>**One intron retention between exons 1 and 2**<br><br>**One intron retention between exons 16 and 17**	**Exon 16**
**Tle6**	**transducin-like enhancer of split 6 (E(sp1) homolog, Drosophila)**	**Exons 5, 6, 9**<br><br>**One intron retention between exons 4 and 5**<br><br>**One intron retention between exons 10 and 11**	**Exon 6**
Slc38a2	solute carrier family 38, member 2	Exons 1-6, 8-14, 16<br><br>Two intron retentions between exons 4 and 5	Intron 3

Dclk1	doublecortin-like kinase 1	Exons 3, 5, 10, 11, 13, 14, 17	Exon 8
Mef2a	myocyte enhancer factor 2A	Exons 3, 4, 5, 7, 8, 10, 11, 12	Exon 6
Sh2b3	SH2B adaptor protein 3	Exons 2, 6-8	Intron 7
Tmem16f	transmembrane protein 16F	Exons 1, 6, 9, 10, 12, 13, 16	Exon 3
Btbd11	BTB (POZ) domain containing 11	Exons 1, 7, 8, 13, 15	Exon 4
Col12a1	collagen, type XII, alpha 1	Exons 1, 2, 4-12, 14-31, 34-41, 43, 49, 50, 53, 55, 57, 58	Exon 13
**Espn**	**espin**	**Exons 15, 16, 18**	**Exon 16**
**Sema3c**	**sema domain, immunoglobulin domain (Ig), short basic domain, secreted**	**Exons 2, 5-12, 15-17**	**Exon 12**
Zeb2	zinc finger E-box binding homeobox 2	Exons 2, 3, 5, 7, 10<br><br>Four intron retentions between exons 2 and 3	Exon 6
**Spint2**	**serine peptidase inhibitor, Kunitz type, 2**	**Exons 1, 3-7**	**Exon 4**
Pde4d	phosphodiesterase 4D, cAMP-specific (phosphodiesterase E3 dunce ho	Exons 6, 8, 9<br><br>One intron retention between exons 3 and 4	Exons 3
Gnb5	guanine nucleotide binding protein (G protein), beta 5	Exons 8, 11, 12	Exon3
**Casp1**	**caspase 1, apoptosis-related cysteine peptidase (interleukin 1, beta, convertase**	**Exons 2,9**	**Intron 2**
Slc27a1	solute carrier family 27 (fatty acid transporter), member 1	Exons 7, 9-12	Exon3
Hsd17b11	hydroxysteroid (17-beta) dehydrogenase 11	Exon 1	Exon 5
**Cd44**	**CD44 molecule (Indian blood group)**	**Exons 8, 11, 13, 19**<br><br>**Two intron retentions between exons 5 and 6**<br><br>**One intron retention between exons 9 and 10**	**Exons 6-15**
Pdgfrb	platelet-derived growth factor receptor, beta polypeptide	Exons 1, 4, 5, 12, 17, 18, 20, 22	Exon 2
Fbxl2	F-box and leucine-rich repeat protein 2	Exons 6, 14<br><br>One intron retention between exons 7 and 8	Exon 12

Sh3rf1	SH3 domain containing ring finger 1	Exons 2, 3, 7, 8	Exon 11
Fgfr1	fibroblast growth factor receptor 1 (fms-related tyrosine kinase 2, Pfeiffer syn	Exons 2,3,5-7, 10, 15, 18	Exons 8, 9
**Myo1b**	**myosin IB**	**Exons 2-4, 7, 10, 15, 17, 20, 24-26, 29**	**Exons 26, 27**
Prss16	protease, serine, 16 (thymus)	Exon 11	Exon 9
Ank3	ankyrin 3, node of Ranvier (ankyrin G)	Exon 15	Exons 2 , 3
Prrx1	paired related homeobox 1	Exons 4, 5	Exon 2
Mllt3	myeloid/lymphoid or mixed-lineage leukemia (trithorax homolog, Drosophila	Exon 5	Exon 9
**Sh3kbp1**	**SH3-domain kinase binding protein 1**	**Exons 12, 20**	**Exon 12**
**Runx1**	**runt-related transcription factor 1 (acute myeloid leukemia 1; aml1 oncogene)**	**Exons 3, 6**	**Exon 3**
Tcf4	transcription factor 4	Exon 12<br><br>Two intron retentions between exons 7 and 8	Exon 10
Tle2	transducin-like enhancer of split 2 (E(sp1) homolog, Drosophila)	Exon 5<br><br>One intron retention between exons 11 and 12	Exon 20
Htatip2	HIV-1 Tat interactive protein 2, 30kDa	Exon3	Exon 2
Stab1	stabilin 1	Exons 9, 12, 34, 38, 47, 49, 50, 52, 67<br><br>One intron retention between exons 42 and 43	Intron 52
Calu	calumenin	Exon 2	Exons 3, 4
Fbxl10	F-box and leucine-rich repeat protein 10	Exon 6	Exon 14
Zwint	ZW10 interactor	Exon 1<br><br>One intron retention between exons 1 and 2	Exon 8
Col1a2	collagen, type I, alpha 2	Exon 12	Intron 31
Uap1	UDP-N-acteylglucosamine pyrophosphorylase 1	One intron retention between exons 1 and 2	Exon 9
**Bcl2l11**	**BCL2-like 11 (apoptosis facilitator)**	**One intron retention between exons 2 and 3**	**Intron 2**
Add3	adducin 3 (gamma)	Exon 12	Exon 16
Col18a1	collagen, type XVIII, alpha 1	Exons 6, 25	Exon 3
Net1	neuroepithelial cell transforming gene 1	Exon 12	Exon 3
**Rai14**	**retinoic acid induced 14**	**Exon 12**	**Exon 12**

Gart	phosphoribosylglycinamide formyltransferase, phosphoribosylglycinamide sy	Exon 9	Exon 11
Spag9	sperm associated antigen 9	Exon 25	Exon 31
Strn3	striatin, calmodulin binding protein 3	One intron retention between exons 8 and 9<br><br>One intron retention between exons 10 and 11	Exons 8, 9
Smarca2	SWI/SNF related, matrix associated, actin dependent regulator of chromatin	Exon 32	Exon 30
Sltm	SAFB-like, transcription modulator	One intron retention between exons 2 and 3<br><br>One intron retention between exons 7 and 8	Exon 6
Mtus1	mitochondrial tumor suppressor 1	Exon 2	Exon 3
St6gal1	ST6 beta-galactosamide alpha-2,6-sialyltranferase 1	Exon 2	Exon 3
**Ttll5**	**tubulin tyrosine ligase-like family, member 5**	**Exons 28-33**<br><br>**One intron retention between exons 30 and 31**<br><br>**One intron retention between exons 32 and 33**	**Exons 33, 34, 35**
**Med 24**	**mediator complex subunit 24**	**Exons 20-26**	**Exon 20**
Hisppd1	histidine acid phosphatase domain containing 1	One intron retention between exons 24 and 25	Exons 26, 27
