# Supplementary material for: Exon-Level Transcriptome Profiling in Murine Breast Cancer Reveals Splicing Changes Specific to Tumors with Different Metastatic Abilities
Source: PLoS One. 2010 Aug 6;5(8):e11981. doi: 10.1371/journal.pone.0011981 (PMC2917353; doi:10.1371/journal.pone.0011981)
Supplement: File S1 — UCSC browser links illustrating probe set level expression differences (fold-change and p-values) for the top 143 isoforms differentially expressed between the samples, obtained from the probe set level analysis. (0.15 MB PDF) [file pone.0011981.s010.pdf]

List of top 143 genes differentially expressed or showing isoform variations between the samples, obtained from the probeset level analysis.

Gene Id	metaprobeset Id	Gene accession	Metaprobeset Anova P-value	Most significant probeset (according to the Anova P-values)	Anova P-value of the most significant probeset	Variation type
Esm1	6810333	NM_023612	3.3623e-07	4381859	1.65878e-10	Gene expression change
Lgals7	6959461	NM_008496	2.6769e-09	4993101	8.31133e-10	Gene expression change
Ttll5	6796707	NM_001081423	0.814311	4463019	1.32039e-09	Alternative termination
Phex	7020407	NM_011077	0.000240667	4772646	2.65678e-09	Alternative termination
Cdh1	6978923	NM_009864	4.15704e-07	4711141	3.62847e-09	Gene expression change
Tspan11	6950030	NM_026743	5.82859e-09	4799240	4.14891e-09	Gene expression change
Mctp2	6968314	NM_001024703	0.473024	5251800	4.25006e-09	Alternative termination
Sorbs3	6825684	NM_011366	8.57759e-09	4990212	5.75107e-09	Gene expression change
Selenbp1	6899622	NM_009150	1.12001e-08	4732701	5.85303e-09	Gene expression change
Slc6a9	6916663	NM_008135	7.03029e-09	4444949	7.21993e-09	Gene expression change

Gpr97	6978355	NM_173036	7.9592e-09	4558899	2.87511e-08	Gene expression change
Galntl2	6818051	NM_030166	3.47877e-07	5579293	3.69854e-08	Gene expression change
Nuak1	6775625	NM_001004363	2.26382e-07	5044705	4.33139e-08	Gene expression change
Med24	6791275	NM_011869	0.822969	4612541	5.14e-08	Alternative termination
3830403N18Rik	7011373	NM_027510	0.430031	5468010	1.01725e-07	Gene expression change
Tmem2	6868650	NM_031997	3.11061e-07	4879286	1.0336e-07	Gene expression change
Cpxm2	6971649	NM_018867	1.83687e-07	4321017	1.41155e-07	Gene expression change
Pdxk	6775258	NM_172134	6.33014e-07	5387636	1.98082e-07	Gene expression change
Acsbg1	6995762	NM_053178	3.01474e-05	5470319	1.98192e-07	Gene expression change
Moxd1	6766605	NM_021509	1.37643e-06	4919017	3.10159e-07	Gene expression change
Tctn2	6934322	NM_026486	7.37958e-07	5175600	3.47676e-07	Gene expression change
Speg	6750628	NM_007463	5.00991e-06	4790184	3.50635e-07	alternative termination
F5	6754691	NM_007976	7.14243e-06	5371468	5.32491e-07	Gene expression change
Gstk1	6945775	NM_029555	2.83576e-06	4711769	5.34862e-07	Alternative termination
Hsp110	6943310	NM_013559	1.11334e-06	4618247	5.9173e-07	Intron inclusion

Rbp1	6991714	NM_011254	3.09675e-07	5149600	7.90461e-07	Gene expression change and additional ASE
Slc23a2	6891065	NM_018824	2.50722e-06	4370578	8.94106e-07	Gene expression change
Mrc2	6784564	NM_008626	2.02111e-05	4538971	1.01778e-06	Alternative initiation
Itgb4	6785183	NM_001005608	5.39913e-05	4832391	1.0794e-06	Gene expression change
Slc39a14	6825688	NM_144808	0.0275794	5087649	1.28125e-06	Cassette exon
Tmcc2	6762217	NM_178874	1.80722e-06	5546043	1.30642e-06	Gene expression change
Lamc2	6763129	NM_008485	1.49146e-05	4633994	1.43859e-06	Gene expression change
Snx30	6913924	NM_172468	3.14787e-05	4751654	1.48953e-06	Gene expression change
Mfi2	6840508	NM_013900	4.78565e-05	5508279	1.91004e-06	Cassette exon
C1s	6957111	NM_144938	1.20949e-05	5186126	1.95327e-06	Gene expression change and additional ASE
Cdcp1	6999645	NM_133974	8.4804e-06	5418062	2.05874e-06	Gene expression change
Odz4	6962736	NM_011858	0.000104455	5039883	2.18442e-06	Gene expression change and additional ASE
Mthfd2	6955025	NM_008638	9.52381e-06	5506990	2.21761e-06	Gene expression change and additional ASE
Abcc3	6790944	NM_029600	6.49554e-05	4576178	2.25481e-06	Gene expression change and additional ASE

Sned1	6751684	NM_172463	1.74323e-05	5329529	2.46575e-06	Gene expression change and additional ASE
Pdk1	6878035	NM_172665	2.06968e-06	5334960	2.99742e-06	Gene expression change
Stch	6847184	NM_030201	1.45334e-07	4925474	4.74918e-06	Gene expression change
Tnfrsf11b	6835728	NM_008764	3.92367e-05	4824202	4.8507e-06	Gene expression change
Lad1	6753400	NM_133664	0.000120839	5280089	5.84337e-06	Gene expression change
St3gal1	6836366	NM_009177	1.06684e-05	5274468	5.86548e-06	Gene expression change and additional ASE
Vdr	6838338	NM_009504	5.4601e-06	5392334	5.89114e-06	Gene expression change
Pam	6760917	NM_013626	0.555705	5594161	7.11915e-06	Cassette exon
Ttyh2	6785079	NM_053273	2.99165e-06	5486011	8.00826e-06	Gene expression change
Mboat1	6805794	NM_153546	4.83752e-05	5230221	8.34649e-06	Gene expression change
Lsp1	6965268	NM_019391	0.0255671	4584904	9.82058e-06	Cassette exon
Cd44	6889258	NM_009851	0.00037452	5425762	1.14058e-05	Several internal cassette exons
Myo1d	6790124	NM_177390	0.000152254	4595636	1.40336e-05	Gene expression change
Mtmr11	6899741	NM_181409	0.00102122	4934563	1.43267e-05	Gene expression change
Epb4.1l5	6761675	NM_145506	0.000208807	4626282	1.58266e-05	Alternative termination

Zeb2	6886356	NM_015753	0.000113227	5270232	2.09246e-05	Gene expression change Intron inclusion
Lbp	6882730	NM_008489	0.000473857	4709873	2.31474e-05	Gene expression change
Itgb1	6980032	NM_010578	0.0881058	5044002	2.42255e-05	Intron inclusion
Acot9	7014702	NM_019736	0.000651868	4655269	2.43886e-05	Intron inclusion
Phb	6783761	NM_008831	0.0148673	5013902	2.68396e-05	Cassette exon
Txlnb	6766250	NM_138628	0.00044298	4390834	2.69322e-05	Cassette exon
Smyd4	6782430	NM_001102611	2.82038e-05	5120524	2.93936e-05	Alternative termination
Copz2	6783873	NM_019877	0.000144699	4431553	3.11904e-05	Gene expression change and additional ASE
Vapa	6856674	NM_013933	0.113335	4394975	3.62092e-05	Intron inclusion
Nmnat2	6754102	NM_175460	9.26754e-06	4861618	3.64019e-05	Gene expression change and additional ASE
Slc25a29	6803596	NM_181328	0.556173	4968317	3.85341e-05	Differential 3' UTR
Naglu	6784237	NM_013792	3.93015e-05	4783626	4.36112e-05	Gene expression changed excepted for one exon
Dmpk	6958984	NM_032418	8.46482e-05	5130757	5.02061e-05	Gene expression change
Angpt2	6980871	NM_007426	0.000833906	4802931	5.24618e-05	Alternative termination and additional ASE
Sh3md4	6768110	NM_172788	2.88638e-06	4752369	5.25524e-05	Alternative initiation
Ier2	6983868	NM_010499	1.35404e-05	4743480	6.07137e-05	Gene expression change

Tmeff1	6913348	NM_021436	0.000358618	4465406	6.46305e-05	Gene expression change and additional ASE
Ccnt2	6752869	NM_028399	0.471421	4910160	7.07365e-05	Intron inclusion
Espn	6927124	NM_207687	8.84641e-05	4607289	7.80303e-05	Gene expression change and additional ASE
Sdha	6814387	NM_023281	0.469836	5202442	8.04116e-05	Intron inclusion
BC035295	6838563	NM_153407	0.0113944	5005900	8.099e-05	Differential 5' UTR
Pdgfc	6898686	NM_019971	0.000527447	5263990	8.7297e-05	Gene expression change and additional ASE
Manba	6901609	NM_027288	0.299147	4979168	8.86985e-05	Intron inclusion
Bclaf1	6766380	NM_001025392	0.554887	4419708	9.15614e-05	Intron inclusion
Hao1	6891210	NM_010403	0.000359901	5524655	0.000123555	Gene expression change and additional ASE
Clip4	6852068	NM_030179	9.62749e-05	5502729	0.000129207	Alternative termination and additional ASE
Ace	6784587	NM_207624	0.000892492	4411818	0.000141381	Gene expression change
Sema7a	6989438	NM_011352	0.000598121	4720501	0.000161687	Gene expression change and additional ASE
Morf4l1	6997712	NM_001039147	0.000240533	4633204	0.000162587	Intron inclusion Cassette exon
Mapk14	6849567	NM_011951	0.430651	4487560	0.000172758	Intron inclusion
Hoxa10	6953615	NM_008263	0.000115064	5138476	0.000176371	Alternative termination

Col15a1	6913269	NM_009928	0.000707639	4603519	0.000190102	Gene expression change
Sat2	6782034	NM_026991	0.000133438	5107368	0.000191057	Cassette exon
Srrt	6942544	NM_031405	0.778046	5382632	0.000195389	Intron inclusion
Gli3	6804974	NM_008130	0.832253	4896933	0.000214804	Intron inclusion
Chid1	6972227	NM_026522	0.00114105	5152830	0.00024234	Intron inclusion
Gosr1	6789825	NM_016810	0.680383	4542236	0.000247966	Intron inclusion
Efna5	6856480	NM_207654	0.759176	4399073	0.000253206	Intron inclusion
Zfp36l2	6857797	NM_001001806	0.000611991	5353460	0.0002769	Intron inclusion
Eny2	6830161	NM_175009	0.426017	5037008	0.000290061	Intron inclusion
Mat2a	6954626	NM_145569	0.000794796	4426868	0.000302479	Intron inclusion
Hprt1	7011264	NM_013556	0.59366	4417069	0.000376608	Cassette exon
Msx1	6937555	NM_010835	0.000165097	4993066	0.000468968	Differential 3' UTR
Cxadr	6842552	NM_001025192	0.0356475	4427200	0.000547345	intron inclusion
Slco3a1	6968426	NM_023908	0.875488	4397959	0.00034695	Intron inclusion
Psd4	6875650	NM_177611	0.859226	5090341	0.000165958	Alternative start
Galnt7	6982695	NM_144731	0.858343	5145519	0.000545445	Intron inclusion
Cdv3	6998401	NM_175565	0.821997	4463201	0.000566789	Intron inclusion
Dlg1	6840507	NM_007862	0.808824	4416637	0.000205605	Intron inclusion
Nfe2l2	6887958	NM_010902	0.789498	4973435	0.000541641	Intron inclusion
Hvcn1	6934119	NM_001042489	0.739488	4830354	0.00041464	Intron inclusion
07-sept	6987580	NM_009859	0.629685	4648373	0.000472546	Intron inclusion
Psmd14	6877587	NM_021526	0.600591	5174667	0.000344707	Intron inclusion
Ewsr1	6785697	NM_007968	0.504199	5386761	0.000533307	Differential 5'UTR
Bicd2	6806938	NM_001039179	0.452181	5057282	0.000271141	Intron inclusion
Itga10	6899767	NM_001081053	0.408871	4758253	0.000452045	Gene expression change
Hist1h1d	6805359	NM_145713	0.250684	5363615	0.000568218	Differential 3'UTR

Pcdh12	6864783	NM_017378	0.315341	5138969	0.000104588	Differential 3'UTR
Luc7l	6849438	NM_028190	0.136604	4710786	0.000233816	Differential 3'UTR
Cdkl2	6939966	NM_016912	0.167998	4385858	0.00045002	Differential 5'UTR
Gpr158	6875564	NM_001004761	0.121855	5434095	0.00031031	Cassette exon
Btg1	6770030	NM_007569	0.379994	5375464	0.000122192	Differential 3' UTR
Amot	7020027	NM_153319	0.116815	4625304	0.000597678	Intron inclusion
2610024G14Rik	6939353	NM_019836	0.10636	4540335	0.000169343	Intron inclusion
4921505C17Rik	6828492	NM_030168	0.0885461	4860945	0.000575568	Intron inclusion
Ercc4	6839491	NM_015769	0.07406	4569089	0.000429462	Intron inclusion
Flrt3	6891493	NM_178382	0.0713676	4495157	0.000525881	Intron inclusion
Srr	6789696	NM_013761	0.0453219	4552498	0.000423413	Intron inclusion
4932441K18Rik	7020636	NM_178935	0.0379941	4305937	0.000350555	Intron inclusion
Nsmce2	6830766	NM_026746	0.0363835	5185915	0.000607018	Intron inclusion
Dusp12	6764033	NM_023173	0.0281431	5323714	0.000530175	Intron inclusion
Dhdds	6925974	NM_026144	0.0207182	5398548	0.000570883	Cassette exon
Tmem19	6777181	NM_133683	0.018245	5041318	0.000177138	Intron inclusion
Btbd3	6881556	NM_145534	0.0160341	5093358	0.000443852	Intron inclusion
Tbc1d23	6846457	NM_026254	0.0158517	5256163	0.000452099	Intron inclusion
Bach1	6842940	NM_007520	0.0136747	4538073	0.0004064	Cassette exon
Dab2	6828472	NM_023118	0.0133713	5009487	0.000605338	Intron inclusion
Shc4	6890611	NM_199022	0.0130502	5268297	0.000263065	Cassette exon
Trabd	6832540	NM_026485	0.0122126	4430185	0.000490147	Intron inclusion
Siah1a	6983978	NM_009172	0.0103497	5375929	0.000635487	Differential 3'UTR
Ccbe1	6866238	NM_178793	0.0098616	4976894	0.000600597	Intron inclusion
Anxa3	6932603	NM_013470	0.00665007	5187641	0.000610686	Intron inclusion
Dcakd	6791656	NM_026551	0.00614967	4753771	0.000533184	Intron inclusion
Il11	6973206	NM_008350	0.00357025	4838543	0.000612932	Cassette exon

Msn	7012681	NM_010833	0.00305852	5161926	0.000447588	Intron inclusion
Stab1	6823709	NM_138672	0.00205131	4604695	0.000269979	Cassete exon, intron inclusion
Pde6d	6760385	NM_008801	0.00178182	4354208	0.000204194	Differential 3'UTR, cassette exon
Hspb7	6918167	NM_013868	0.00144202	4352925	0.000527172	Differential 3'UTR
Dlgap4	6882622	NM_146128	0.0011047	5337902	0.000387847	Intron inclusion
