# Supplementary material for: Exon-Level Transcriptome Profiling in Murine Breast Cancer Reveals Splicing Changes Specific to Tumors with Different Metastatic Abilities
Source: PLoS One. 2010 Aug 6;5(8):e11981. doi: 10.1371/journal.pone.0011981 (PMC2917353; doi:10.1371/journal.pone.0011981)
Supplement: File S2 — UCSC browser links illustrating the probe set level expression differences (fold-change and p-values) as well as the normalized (SI) differences for the top 60 isoforms differentially expressed between the samples, obtained from the Splicing Index analysis. (0.10 MB PDF) [file pone.0011981.s011.pdf]

List of top 63 genes showing isoform variations between the samples, obtained from the Splicing Index analysis.

Gene Id	metaprobeset Id	Gene accession	Metaprobeset Anova P-value	Most significant probeset (according to the Anova P-values)	Anova P-value of the most significant probeset	Variation type
Rpl23	6791222	NM_022891	0.0171427	5299095	0	Intron inclusion
Supv3l1	6774395	NM_181423	0.13222	4577996	2.57875e-06	Intron inclusion
Nudcd3	6785762	NM_173748	0.0121992	5104326	3.28722e-06	Intron inclusion
Arg1	6772815	NM_007482	0.013858	5234845	4.52314e-06	Intron inclusion
Agrn	6927362	NM_021604	0.871173	5131440	1.12815e-05	Intron inclusion
Nfib	6922846	NM_008687	0.0280349	4744985	3.11013e-05	Cassette exon
Slc1a5	6958905	NM_009201	0.898088	5215935	3.57773e-05	Intron inclusion
Trim26	6850250	NM_001025599	0.628829	4376250	3.70235e-05	Intron inclusion
R3hdm1	6752884	NM_181750	0.118452	5123092	5.88997e-05	intron inclusion
Zfand5	6868618	NM_009551	0.624094	4842770	5.96966e-05	Intron inclusion
Clk1	6758941	NM_001042634	0.0193742	4418371	8.31211e-05	Intron inclusion
4121402D02Rik	6784048	NM_028722	0.427162	4688500	8.51614e-05	Intron inclusion
Il18r1	6748889	NM_008365	0.064024	4892072	8.61301e-05	Intron inclusion
Satb2	6758862	NM_139146	0.0713463	5305589	9.90311e-05	Intron inclusion
Nisch	6823710	NM_022656	0.898708	4741010	0.000100809	Intron inclusion
Ivns1abp	6754014	NM_054102	0.245809	4657092	0.000101616	Intron inclusion
Fmr1	7011757	NM_008031	0.660569	4730469	0.000141621	Cassette exon
Rsrc2	6941837	NM_025438	0.80143	5453603	0.000148816	Cassette exon
Rap1gds1	6909871	NM_001040690	0.631144	4953216	0.000151893	Intron inclusion
Plekha3	6878347	NM_031256	0.0428608	5410024	0.000155166	Cassette exon

Hsf1	6831660	NM_008296	0.404479	4687158	0.000160432	Intron inclusion
Hisppd1	6760915	NM_173760	0.946155	5045686	0.000166998	Intron inclusion
Npr2	6913020	NM_173788	0.00103108	4539803	0.000180028	Intron inclusion
Stag1	6991873	NM_009282	0.424899	4958415	0.000190372	Intron inclusion
Ehd1	6867849	NM_010119	0.159099	4756891	0.000195054	Intron inclusion
Zfp410	6796595	NM_144833	0.589022	5014929	0.000209162	Intron inclusion
Sesn1	6767402	NM_001013370	0.891209	5327098	0.000216404	Intron inclusion
Lrrc8d	6933141	NM_178701	0.0526712	4313723	0.000258536	Intron inclusion
Hnrph1	6780797	NM_021510	0.989222	4365203	0.000267263	Intron inclusion
Ift172	6937081	NM_026298	0.0233091	4979678	0.000292452	Alternative termination
Wdtc1	6925885	NM_199306	0.0661967	5207947	0.00029357	Intron inclusion
Rbm9	6836888	NM_053104	0.0477287	4687323	0.000298695	Intron inclusion
Gdpd3	6964245	NM_024228	0.0169311	4963283	0.000317246	Cassette exon
Rai14	6833980	NM_030690	0.0130151	4456110	0.000327353	Cassette exon
Tm4sf5	6782129	NM_029360	0.0160368	5569949	0.000354744	Cassette exon
Slk	6870166	NM_009289	0.15608	5355354	0.000745324	Cassette exon
Lace1	6773546	NM_145743	0.00851393	5219952	0.000506945	Intron inclusion
H3f3b	6792486	NM_008211	0.00862016	5019598	0.000660606	Intron inclusion
Thop1	6769262	NM_022653	0.0166104	5063925	0.00058844	Intron inclusion
Parp11	6949992	NM_181402	0.017502	4681790	0.000474618	Intron inclusion
Huwe1	7014551	NM_021523	0.0302209	4469179	0.000265265	Cassette exon
Mapk8ip3	6854400	NM_013931	0.0335469	5051055	0.000536173	Intron inclusion
Rbm39	6892423	NM_133242	0.0418497	5241837	0.000606504	Intron inclusion
Paip1	6810620	NM_145457	0.0863144	4766267	0.000495694	Differential 3'UTR
Vps37a	6975614	NM_033560	0.0978693	5206157	0.000272275	Intron inclusion

Pfdn4	6883526	NM_001013369	0.144583	4768708	0.000450425	Intron inclusion
BC037034	6942617	NM_153161	0.168246	4946452	0.000604411	Intron inclusion
Rfwd2	6754437	NM_011931	0.188957	4974623	0.000587991	Intron inclusion, cassette exon
Aldh1a7	6872029	NM_011921	0.196089	5564677	0.000587049	Cassette exon
Arid1a	6925936	NM_001080819	0.235644	5224087	0.000562195	Intron inclusion
Drg1	6785591	NM_007879	0.274161	4403628	0.00050447	Intron inclusion
Nfkbiz	6846375	NM_030612	0.372406	5259270	0.000701173	Intron inclusion
Zfr	6828790	NM_011767	0.410785	4418390	0.000691603	Intron inclusion
Golga4	6992852	NM_018748	0.438378	4311969	0.000527865	Intron inclusion
Sirt7	6792787	NM_153056	0.491175	5164019	0.000422599	Intron inclusion
Tkt	6817930	NM_009388	0.623445	5035917	0.000514145	Intron inclusion
Npepps	6791175	NM_008942	0.683337	4920034	0.000676699	Intron inclusion
Krit1	6928479	NM_030675	0.878792	4642200	0.000627976	Intron inclusion
Eif4g1	6839956	NM_145941	0.886587	4779060	0.000611052	Intron inclusion
Golga1	6886200	NM_029793	0.908608	4922440	0.000483053	Intron inclusion
